# Author Correction: Adaptive mechanisms of social and asocial learning in immersive collective foraging

**DOI:** 10.1038/s41467-025-61159-5

**Published:** 2025-06-20

**Authors:** Charley M. Wu, Dominik Deffner, Benjamin Kahl, Björn Meder, Mark K. Ho, Ralf H.J.M. Kurvers

**Affiliations:** 1 Human and Machine Cognition Lab, University of Tübingen, Tübingen, Germany; 2 Centre for Adaptive Rationality, Max Planck Institute for Human Development, Berlin, Germany; 3 Department of Computational Neuroscience, Max Planck Institute for Biological Cybernetics, Tübingen, Germany; 4 Excellence Cluster: Science of Intelligence, Technical University Berlin, Berlin, Germany; 5 Department of Psychology, University of Marburg, Marburg, Germany; 6 Institute for Mind, Brain and Behavior, Department of Psychology, Health and Medical University, Potsdam, Germany; 7 Department of Psychology, New York University, New York, NY USA

**Keywords:** Human behaviour, Decision making

Correction to: *Nature Communications* 10.1038/s41467-025-58365-6, published online 25 April 2025

In this article the author name ‘Mark K. Ho’ was incorrectly written as ‘Mark H. Ho’. The original article has been corrected.

