# Supplementary material for: Adaptive mechanisms of social and asocial learning in immersive collective foraging
Source: Nat Commun. 2025 Apr 25;16:3539. doi: 10.1038/s41467-025-58365-6 (PMC12032219; doi:10.1038/s41467-025-58365-6)
Supplement: Supplementary file 2 — Reporting Summary [file 41467_2025_58365_MOESM2_ESM.pdf]

nature portfolio

Corresponding author(s): Charley M. Wu

Last updated by author(s): Dec 26, 2024

Reporting Summary

Nature Portfolio wishes to improve the reproducibility of the work that we publish. This form provides structure for consistency and transparency in reporting. For further information on Nature Portfolio policies, see our Editorial Policies and the Editorial Policy Checklist.

Statistics

For all statistical analyses, confirm that the following items are present in the figure legend, table legend, main text, or Methods section.

n/a	Confirmed	
☐	☒	The exact sample size (*n*) for each experimental group/condition, given as a discrete number and unit of measurement
☐	☒	A statement on whether measurements were taken from distinct samples or whether the same sample was measured repeatedly
☐	☒	The statistical test(s) used AND whether they are one- or two-sided<br>*Only common tests should be described solely by name; describe more complex techniques in the Methods section.*
☐	☒	A description of all covariates tested
☐	☒	A description of any assumptions or corrections, such as tests of normality and adjustment for multiple comparisons
☐	☒	A full description of the statistical parameters including central tendency (e.g. means) or other basic estimates (e.g. regression coefficient) AND variation (e.g. standard deviation) or associated estimates of uncertainty (e.g. confidence intervals)
☐	☒	For null hypothesis testing, the test statistic (e.g. *F*, *t*, *r*) with confidence intervals, effect sizes, degrees of freedom and *P* value noted<br>*Give P values as exact values whenever suitable.*
☐	☒	For Bayesian analysis, information on the choice of priors and Markov chain Monte Carlo settings
☐	☒	For hierarchical and complex designs, identification of the appropriate level for tests and full reporting of outcomes
☒	☐	Estimates of effect sizes (e.g. Cohen's *d*, Pearson's *r*), indicating how they were calculated

Our web collection on statistics for biologists contains articles on many of the points above.

Software and code

Policy information about availability of computer code

Data collection	Data collection was conducted using a custom mod developed for Minecraft Java Edition ver. 1.12.2 using Forge ver. 14.23.5.2847. The code has been published here https://github.com/charleywu/minecraftforaging
Data analysis	Data analysis was conducted using R 4.3.0 and Python 3.7.4. The visual-field transcription was performed using custom software as a standard Unity project using Unity version 2019.3.0f5 with no additional dependencies. Statistical models were fit using the Stan MCMC engine via the rstan (2.21.2) and cmdstanr (0.5.3) packages. All code used to produce the analyses have been published here https://github.com/charleywu/minecraftforaging

For manuscripts utilizing custom algorithms or software that are central to the research but not yet described in published literature, software must be made available to editors and reviewers. We strongly encourage code deposition in a community repository (e.g. GitHub). See the Nature Portfolio guidelines for submitting code & software for further information.

Data

Policy information about availability of data

All manuscripts must include a data availability statement. This statement should provide the following information, where applicable:

- Accession codes, unique identifiers, or web links for publicly available datasets
- A description of any restrictions on data availability
- For clinical datasets or third party data, please ensure that the statement adheres to our policy

All data collected in the experiment and used for performing the analyses reported in the paper have been uploaded here: https://github.com/charleywu/minecraftforaging

Research involving human participants, their data, or biological material

Policy information about studies with human participants or human data. See also policy information about sex, gender (identity/presentation), and sexual orientation and race, ethnicity and racism.

Reporting on sex and gender	128 participants participated in the study with 82 self-identifying as women. The rest identified as males, but a free text field was available for other gender identities. No sex or gender-based analyses were performed because there were no a priori theoretical reasons with respect to the research question. Thus, the terms "sex" and "gender" are not used in the paper, but the self-reported gender identifies have been included in the open data for interested researchers.
Reporting on race, ethnicity, or other socially relevant groupings	We do not report nor did we collect data on race, ethnicity or class in the manuscript, since they were not relevant for our research questions.
Population characteristics	We restricted the age of invited participants to 18-50 because we did not want to introduce variability in experience with computers and games. Participants were on average 27.4 ± 5.0 (SD).
Recruitment	Participants were invited via email from the Max Planck Institute for Human Development (MPIB) recruitment pool in Berlin. Thus, we may have a recruitment bias from people more likely to use a computer.
Ethics oversight	The study was approved by the Institutional Review Board of the MPIB (number: A 2019-05) and participants signed an informed consent form prior to participation

Note that full information on the approval of the study protocol must also be provided in the manuscript.

Field-specific reporting

Please select the one below that is the best fit for your research. If you are not sure, read the appropriate sections before making your selection.

☐ Life sciences ☒ Behavioural & social sciences ☐ Ecological, evolutionary & environmental sciences

For a reference copy of the document with all sections, see nature.com/documents/nr-reporting-summary-flat.pdf

Behavioural & social sciences study design

All studies must disclose on these points even when the disclosure is negative.

Study description	Quantitative experimental study using visual, spatial and action data from participants in a virtual minecraft environment
Research sample	Our participants were recruited from an institute participant pool (see above for details) and can be considered a convenience sample The sample is more diverse in terms of age and social-economic status than a typical student sample, but may suffer from being biased towards Westernized, Industrialized, Educated, Rich, and Democratic (WIERD) societies. Thus, future studies should investigate whether our analyses hold in more diverse cultures.
Sampling strategy	Random sampling was employed to reach a target sample size of 128 participants. This sample size was based on previous pilots of the experiment (incl. Wu et al., CogSci 2019), and facilitated an even number of between group pseudo-randomizations of round order. However, all main manipulations were within subject (environment and solo vs. group).
Data collection	Data was collected in the behavioral experiment lab at the MPI for Human Development in Berlin. The study was conducted by a lab manager, who observed the data collection in an adjacent room but was blinded to the aim of the study and the hypotheses. Participants were seated in the same room with opaque divider panels ensuring they could not observe each other's monitors. All sounds were disabled from the experiment.
Timing	Data was collected between November 3rd and December 10th, 2021

Data exclusions	No data was excluded
Non-participation	No participants dropped out
Randomization	Groups of participants were randomly allocated to a pre-generated pseudo-randomization of manipulation order, where we manipulated environment (random vs. smooth) and condition (solo vs. group) within subject. The mapping of environment to pumpkin vs. watermelon blocks was also counterbalanced across groups using the same pseudo-randomization.

Reporting for specific materials, systems and methods

We require information from authors about some types of materials, experimental systems and methods used in many studies. Here, indicate whether each material, system or method listed is relevant to your study. If you are not sure if a list item applies to your research, read the appropriate section before selecting a response.

Materials & experimental systems

n/a	Involved in the study
☒	☐ Antibodies
☒	☐ Eukaryotic cell lines
☒	☐ Palaeontology and archaeology
☒	☐ Animals and other organisms
☒	☐ Clinical data
☒	☐ Dual use research of concern
☒	☐ Plants

Methods

n/a	Involved in the study
☒	☐ ChIP-seq
☒	☐ Flow cytometry
☒	☐ MRI-based neuroimaging

Plants

Seed stocks	N/A
Novel plant genotypes	N/A
Authentication	N/A
