# Supplementary material for: Adaptive mechanisms of social and asocial learning in immersive collective foraging
Source: Nat Commun. 2025 Apr 25;16:3539. doi: 10.1038/s41467-025-58365-6 (PMC12032219; doi:10.1038/s41467-025-58365-6)
Supplement: Supplementary file 3 — Transparent Peer Review file [file 41467_2025_58365_MOESM3_ESM.pdf]

nature portfolio

Peer Review File

Adaptive mechanisms of social and asocial learning in immersive collective foraging

Corresponding Author: Dr Charley Wu

This file contains all reviewer reports in order by version, followed by all author rebuttals in order by version.

Version 0:

Reviewer comments:

Reviewer #1

(Remarks to the Author)
Overall, this is very nice work, with an experiment in a virtual foraging environment showing different foraging strategies for smooth (clumpy) and random resource distributions, as well as a difference in asocial and social environments. When foragers were in social environments, they used social cues in the smooth environment when their own performance was poor and other foragers were doing well. The authors use a model competition to identify an area-restricted search strategy that is also conditionally social as the best strategy in smooth and social environments.

I have very few comments on this work as I think it is already in excellent shape.

1. I recall some additional work by Goldstone and Roberts that provides a model of social foraging, EPICURE, and several virtual social foraging experiments. The results provided here are consistent with those, somewhat, but it might be worth discussing them in context to draw out some of the (dis)similarities. Roberts, M. E., & Goldstone, R. L. (2006). EPICURE: Spatial and knowledge limitations in group foraging. Adaptive Behavior, 14(4), 291-313. Goldstone, R. L., Ashpole, B. C., & Roberts, M. E. (2005). Knowledge of resources and competitors in human foraging. Psychonomic Bulletin & Review, 12(1), 81-87.
2. Social learning can be maladaptive if foraging is destructive as one will simply travel to a location where others have already harvested resources, this is especially true in the random environments. As I understand the results, people were somewhat pulled even in random environments. What might be the explanation here?
3. One explanation is that people did not know that the resources were or were not clustered and this took time to detect? Is there evidence of learning within an environment?
4. For future work, one could investigate anti-correlated spatial distributions, where targets are not near other targets. Then social learning would be at its worst.
5. The authors might want to connect this to the group problem solving literature. Goldstone, R. L., & Gureckis, T. M. (2009). Collective behavior. Topics in cognitive science, 1(3), 412-438. Barkoczi, D., & Galesic, M. (2016). Social learning strategies modify the effect of network structure on group performance. Nature communications, 7(1), 13109.
6. Related to the above, Figure 2a might be interpreted to suggest that social groups are really no better than groups of individuals in this world---harvest rates aren't higher. Perhaps there is a better way to show that, if it's true. That might be related to a notion in Giraldeau, L. A., & Caraco, T. (2000). Social foraging theory (Vol. 73). Princeton University Press, which points out that social groups can often be no better than individuals because as long as they are better, new individuals will join them until the benefits of social foraging are equivalent to solo foraging.
7. The authors might want to point out the evolutionary generalizability of area-restricted search. This isn't entirely relevant on the face of it, but note that area-restricted search can easily be generalized to 'social' resources. People can perseverate on social evidence of resource collection, even when they are not receiving it themselves. This merely requires a method for attaching social evidence of resources to 'reward detection'. I think this complicates and enhances the idea of area-restricted search and goal-directed cognition I set out here: Hills, T. T. (2006). Animal foraging and the evolution of goal-directed cognition. Cognitive science, 30(1), 3-41. Note in that work that I talk about the evolution of the area-restricted search system to talk about the "expectation of a reward", which can lead to local search around the expectation.

Thomas Hills

(Remarks on code availability)
I looked over the code but did not run it. It seems well commented and fairly clear.

Reviewer #2

(Remarks to the Author)

The paper developed a novel Minecraft-inspired collective foraging experiment in an immersive environment to investigate the mechanisms behind adaptive social learning. The task creates a realistic situation in capturing human social foraging behavior and the task design can be an important contribution to the field. The study replicates findings from previous models and theories about foraging behavior and the use of social learning strategies (eg, success biased copy, performance adaptive copying). Although we like the task design, we believe that the motivations behind it and then analyses done leave a few questions unanswered, and some conclusions are not well-supported by the analyses. Mainly, the lack of "adaptive" nature of social learning in this task and comparisons between solo and group foraging, and the general unification of social and asocial learning raise questions.

General comments:

1.General framework and motivations: The paper's main motivation is pitched as broad implications for social learning, i.e., what kind of cues underlie adaptive social learning in a foraging context and there is an attempt to distinguish between asocial and social cues, and to unify asocial and social learning in a broad manner. Why is an immersive Minecraft style experiment required to study this? The authors say that the virtual environment poses a limited field of view which creates a natural trade-off between individual search and social learning. Sure, it creates a natural trade-off, but why should we care about visual-spatial dynamics in social learning? For example, in the abstract, the authors start with talking about adaptation to different environments and adaptive behavior and highlight that an integrated framework is missing. They then jump directly to describing the Minecraft environment and the visual field and spatial data they gain from it. How are visual and spatial data related to adaptive behavior in social and asocial context? Why does visual attention matter?

2. "Adaptive social learning": The paper claims that visual spatial information drives adaptive social learning. But at group level, there is no difference in normalized reward rate between solo and group condition. Thus it seems that providing social information does not increase individual foraging performance. Because a person can not individually explore and learn socially at the same time, it is natural for people to switch between social and asocial learning in the group condition compared to solo condition. It remains unclear what is adaptive about social learning. We talk about this point more in the section on Results.

3. Solo vs social: The authors compared between as-if solo condition and group condition in many (fig a-f) but not all (fig g-i) analyses. We believe some analyses lack controls. In this task, all participants have the same goal and thus in the smooth environment people would gather at the same rewarding location no matter if they were independently exploring or following others. Thus, it is critical to show what effect is driven by social learning and not the environment structure. Please see the later sections for detailed opinions.

4. "Unifying asocial and social":
a)The paper starts with an ambitious goal of unifying social and asocial theories of learning and decision-making but while the computational model combines social information and personal information, it does not really unify it meaningfully. Many past studies have shown that people balance between social and asocial information (eg Tump et al 2020, Toyokawa et al 2019) and many models have done so to, so what is this paper's contribution to the "unification"?

b)In addition, the authors refer to ARS for asocial adaptive strategy and critical/conditional social learning as social adaptive strategy and pit them against each other, while they are not really opposing strategies. The basic mechanism of ARS or general optimal foraging theory is related to adapting search state based on the rate of current rewards (or expectation of future rewards). Same goes for critical/conditional social learning, which is based on switching between asocial or social strategy based on reward rate of self (and social partner). They both hinge on the same mechanism, but the only difference lies in where the information about reward is coming from. ARS might as well be called "success-biased" asocial learning because it is biased towards success.

Results:

1. A basic expectation from social learning would have been that players are better at finding rewards with social information. But that is not the case, there is no reliable difference between solo and social condition in the normalized reward rate provided here. What did the overall score look like per participant? Supplementary results say that for smooth environments, participants performed equally well in solo and group condition in terms of normalized reward rate. So how is this adaptive social learning? If there is adaptive social learning indeed, there should be an increase in at least the rate of

finding rewards, or in the ratio of reward blocks found / total blocks destroyed. What is the extent of space covered by the participants? If there is no difference in the space covered between social and solo conditions, i.e., they cover the same ground then maybe it can explain why there is no difference in reward rate.

2. The authors found that there are no visual differences in the smooth environment between as-if solo and group conditions, but participants reduced social visibility in random environments. This seems to suggest that people use visual spatial information to avoid competition rather than social learning. In addition, the predictor in the regression is undirectional pairwise distance, but later analyses use directional data. So why not separate in-degree and out-degree in this regression analysis?

3. “As-if” solo rounds: a)Can authors clarify how the as-if rounds were constructed? The task structure is a little confusing - there were 16 rounds in the main task, were they all on different environment structures? Or were there 4 randomly picked environments for each condition (solo vs group, random vs smooth)? Or were there 2 randomly picked environments for random vs smooth, and participants played both solo and group conditions on just these two environments. The “as-if” rounds will only make sense if the environment structure was completely identical between solo and group (for a given resource condition, that is). If that is indeed the case, is there any effect of learning or memory?

b) Plus, why should there be any expectation of meaningful visibility/proximity networks in the as-if models? Better analyses would be to compare how individual foraging behavior changes when in groups – for example through ARS etc. In the smooth environment, there seems to be clear clusters of rewards so do players aggregate on a cluster? One can imagine a cluster of blocks to be a patch and then compare their patch leaving time in solo vs group rounds. In solo foraging, it will be solely based on their “asocial cue” i.e., reward rate, while in group, it will be based on self’s reward rate and others’ reward rate.

c) It is unclear whether the effects reported here are simply due to the task structure or due to social adaptations. For example, average proximity is higher in smooth rounds than in random rounds. There is a higher likelihood for people to end up on the same cluster of blocks. In addition, due to the solo rounds, they know whether to expect more rewards nearby previous rewards or not based on the color of the blocks. So irrespective of social adaptations or not, they will overlap or not overlap based on the foraging expectations.

4. Proximity and visibility networks:
a) Are the network weights static and averaged over the whole experiment or are they dynamic?
b) Visibility networks: The authors found an asymmetry between the in- and out-degree are more in the group+smooth sections. How does an inverse relationship suggest "specialization of social learning strategies"? What is the strategy that is being discussed and what does it mean for it to be specialized? This effect can also be driven by the spread of players, if there are some players who are more ahead of others or moving in a different direction then others can see them but they can’t see others much. Alternatively, this could also be driven by the fact that in general some participants looked more in the group section and thus other participants got more social attention. This would explain the effect of out- and in-degree in random environments and not just in smooth. In smooth, this would lead to a stronger effect because most people are localized near each other and the few people who are away from the rest of the group will have a high in-degree. In random, because people are dispersed and not synchronized the effect is weaker.

5. Temporal analyses:
a)The authors need to better explain the clusters and correlations and offsets, it is hard to follow. For example, what are “chance corrections”? What are predictive clusters and how were they chosen?

b)Again, why will the as-if condition show any effect of proximity and reward temporality? A better comparison would be to see what individual players do when they have low reward rate and high reward rate in solo vs group. If the behavior (movement, head turns or something) is different between solo and group, only then there would be clear proof that players socially adapted the strategy. The authors mention ARS but then it is only discussed in the computational results. The “performance-adaptive-social-cycling” is attributed to social adaptation: poor rewards lead to increased future proximity and high rewards lead to decreased future proximity. While we agree that people follow a player who has found something, the “social-cycling” need not be purposeful – the destructive blocks will inadvertently lead to that effect because after exhausting the rewards in an area (high rewards), the strategy can be to move away from that area (like in ARS) and proximity reduction will be a side-effect. Then when they see someone else find a reward, they converge to a local area. Is there a social and asocial comparison to get at the behavior?

c)Similarly for adaptive social attention, low rewards predicted seeing more peers in the immediate future and vice-versa, this can be very well driven by the destructive structure and the expectation of reward structure which will lead them to move away or look away after destroying enough rewards in the local area. How do head turns or rotations in social rounds compare with solo because that is the meaningful comparison.

6. Computational modeling:
a)The comparisons between behavioral results and the model seem disjointed. We’d like to see more connections of the model to the behavioral results (eg, what distinctive behavioral patterns do different models predict?) and other visibility analyses (eg, does the model predict more pull behavior / more in degree / ect?). In addition, ARS was used in the model but

there is no discussion in the behavioral results.

b)The winning model combines ARS and conditional social learning, which hints at the precedence of individual success rate in deciding the next step. However, this may not be as general an effect as the paper makes it out to be. In environments where individual search is costlier or when there are no prior expectations from the environment, critical social learning would be more adaptive. The effect of task design in the “general” features of adaptive behavior need to be specified.

Other minor comments:
a)The authors wrote “Whereas high centrality tended to correspond to low rewards in random environments (group: -7.6 [-16.6,0.6]; solo: -7.6 [-16.1,0.8]; all slopes overlap with 0)”. Although the authors used the word “tended”, I don’t think they can draw any conclusion from a confidence interval overlapping with 0.

b)The introduction can be better set up. Many terms are dropped in without context or explanation of what they mean. For example, what social or asocial cues or factors should we expect in the following sections? What does “influence and selectivity” of social learning mean?

c)The papers cited in the first line of Discussion do not really refer to collective foraging as a metaphor for social learning to my knowledge.

(Remarks on code availability)

Reviewer #3

(Remarks to the Author)
I co-reviewed this manuscript with one of the reviewers who provided the listed reports. This is part of the Nature Communications initiative to facilitate training in peer review and to provide appropriate recognition for Early Career Researchers who co-review manuscripts.

(Remarks on code availability)

Reviewer #4

(Remarks to the Author)
NCOMMS-24-13967
Visual-spatial dynamics drive adaptive social learning in immersive environments

Upfront, I’m a statistician with no expertise in the content area of the paper. My area of expertise is hierarchical modeling and I was asked to review those aspects of the paper. To be frank, although I am passively familiar with agent-based modeling and network modeling, I don’t have much expertise in this area and am not familiar with nuances and best practices, so I am not sure how relevant or informative my review will be.

That said, I thought the detail on the hierarchical models looked pretty good. I thought the number of MCMC iterations (4,000) looked a little low for this kind of model, but the authors did a convincing job reporting convergence/diagnostic information on p. 13 to demonstrate that the results are reasonably trustworthy. The models were also described completely and it was generally clear to me how things were set up.

As a moderately important comment, the description on p. 11 says the random effects in the model control for participant characteristics. This is a common misconception, but it is not actually true. The random effects serve to partition the unexplained variance into different sources (e.g., within-unit vs between-unit), but the variance is still unexplained and participant characteristics are not conditioned out. If you truly wanted to control for participant characteristics, you’d have to use a fixed effect approach (e.g., include dummy variables for each unit) to absorb the participant sources of variance or you’d have to center the predictors in a way that make the within-unit and between-unit submodels orthogonal such that participant characteristics could not affect within-unit associations (e.g., a within-between specification where within-unit predictors are unit-mean centered and the unit-mean is included as a between-unit predictor; e.g., Bell & Jones, 2015, Political Science Research & Methods; McNeish & Kelly, 2019, Psychological Methods). To be clear, I’m not saying that the model is wrong or inappropriate, just that I don’t believe the description matches what the model is doing.

As some minor comments, it’d be helpful to report the argument for the LKJ prior (typically referred to as eta). I’m guessing it was 1 since that is commonly used so that the prior is a random correlation matrix of a particular dimension, but different arguments for LKJ correspond to correlation matrices with different properties that may not necessarily be diffuse/non-informative.

Additionally, CIs are mentioned in a few places but it is not defined (confidence intervals or credible interval? CI can be used for both). If credible interval, it would be useful to mention what kind of interval (e.g., symmetric, highest posterior density). P.2 mentions highest density intervals, but it wasn't clear if this referred to just those values or to any CI throughout the paper and in the figures.

(Remarks on code availability)

Reviewer #5

(Remarks to the Author)
The paper aims to elucidate the mechanisms of social and asocial learning within a unified framework. The study integrates both non-social and social learning strategies within a virtual Minecraft environment, where participants engage in collective foraging tasks. Automated transcription of visual field data and high-resolution spatial trajectories are used to analyze social interaction dynamics. The paper demonstrates that people dynamically adapt their non-social and social learning mechanisms based on environmental conditions and personal performance, specializing their social attention patterns and selectively directing social learning toward successful individuals. Additionally, the study introduces a computational modelling framework that integrates various combinations of non-social and social learning mechanisms to predict individual foraging decisions sequentially.

The primary strength of this paper lies in its practical experimental framework, which allows for a comparative analysis of social and asocial learning. The use of a Minecraft-based foraging game is particularly clever, as it enables participants to engage in both learning strategies and provides a rich experimental setup that mirrors real-world social interactions. This framework facilitates dynamic analysis of decision-making processes at each time point. By quantifying abstract human learning strategies through experimental results, the study establishes a solid foundation for future experimental modelling. Incorporating computational models adds depth to the behavioural analysis, offering a new understanding of cognitive processes. This methodological approach provides significant insights and inspiration for future research, demonstrating a multidimensional strategy that could have a profound impact on social science studies.

However, several concerns need to be addressed before the paper can be recommended for publication.
1. Theoretical Contributions: The paper's theoretical contributions are not clearly articulated. The experimental results are somewhat broad and scattered, making it unclear what key scientific question the authors are addressing. Are they attempting to show that individuals can balance social and asocial learning based on their goal of maximizing personal performance? This may not be a novel finding, as from an evolutionary perspective, striving for optimal performance is a natural expectation for enhancing foraging efficiency. It gives the impression that, despite the authors employing new designs and conducting extensive data analyses and modeling, these efforts have not yielded new scientific insights for the field. In other words, the authors should clarify how their approach addresses gaps left by existing theoretical and experimental frameworks and what significant theoretical conflicts have been resolved through their methodology.

2. The purpose of analyzing leadership: While the analysis of leadership effects on social learning is interesting, its relevance to the paper's main argument—that individual performance drives the adaptivity of both local foraging and selective social learning—is unclear. The implications of leadership positions on social attention and behaviour are not well-integrated into the core thesis. This aspect might be more appropriately explored as a separate study to better understand its significance and impact.

3. Gaze vs. Heading: The paper should distinguish between "gaze" and "heading." In real-world scenarios, gaze and heading are not always aligned due to differences in eye orientation, head direction, and body orientation. Since participants in the study used a screen and mouse and keyboard controls, their gaze and heading are likely to be consistent, which does not fully reflect real-world conditions. Although the authors chose this approach to avoid VR motion sickness, it may lead to discrepancies between participants' actual walking trajectories and the avatars' movements. Given that data collection likely began in 2019 (as indicated by the ethics approval number) and considering the advancements in VR technology over the past five years, including improvements that mitigate motion sickness, modern all-in-one VR headsets now allow users to walk freely in an unlimited virtual space. Incorporating this technology could significantly enhance the naturalness of the experiment by enabling participants to walk within the virtual environment while interacting with the game. I recommend that the authors utilize VR headsets that support free movement in virtual spaces to improve the experiment's realism and potentially achieve more accurate results.

4. Concept of Social Interaction: The paper should clarify what is meant by "social interaction," as it is a broad concept. It appears to use an abstract notion rather than specifying whether it refers to direct face-to-face interaction or verbal communication. The authors should clearly define what "social interaction" specifically represents in their study.

5. Data Analysis Justification: Given the complexity of the data analyses, the authors should provide more details at the beginning of each results section about the necessity of each analysis. This includes explaining the rationale behind each analysis, the hypotheses being tested, and the insights that the results reveal.

In sum, while the paper presents a sophisticated experimental design and innovative methodology, it needs to clarify its theoretical contributions and ensure that all aspects of the research, including leadership analysis and technology choices, are well-justified and contribute to the main thesis. Addressing these concerns will strengthen the paper and its impact on

the field of social and asocial learning research.

(Remarks on code availability)
The code includes a comprehensive README file with detailed instructions for installation and running the application.

Version 1:

Reviewer comments:

Reviewer #1

(Remarks to the Author)
I'm very satisifed with the revision. It's sophisticated and enlightening and demonstrates a nice potential interaction between social and asocial area-restricted search. The next step will be to see if they share underlying mechanisms. How nice it would be to show that the evolution of spatial search is somehow related to social learning and mental search more generally, and perhaps shares fundamental dimensions.

(Remarks on code availability)

Reviewer #2

(Remarks to the Author)
We thank the authors for addressing our comments, especially by tying their motivations closer to the experiment and results. We agree that the integration of asocial foraging and social information is novel, and this new virtual foraging paradigm is a good way to study this question. The comparison with as-if solo rounds is also explained more clearly in this revision.

We only have two minor comments:
1. The authors started from the assumption that these are adaptive and showed that people employed these computations in social foraging, and employed them differently in different environments. And the computational models provide a proof for this assumption. The authors also showed that greater adaptive weights predict better performance in social foraging. We think this is a major finding supporting the main motivation and title, and could be emphasized more.

2. The authors also talked about how people with greater centrality performed better, and leaders achieved higher instantaneous reward. But these are effects instead of mechanisms. We hope these could be tied more to the main motivation, or addressed in the Discussion.

(Remarks on code availability)

Reviewer #3

(Remarks to the Author)
I co-reviewed this manuscript with one of the reviewers who provided the listed reports. This is part of the Nature Communications initiative to facilitate training in peer review and to provide appropriate recognition for Early Career Researchers who co-review manuscripts.

(Remarks on code availability)

Reviewer #4

(Remarks to the Author)
I was Reviewer 4 on the original submission. All of my comments related to the statistical analyses were satisfactorily addressed and I have no further comments on this version of the paper.

(Remarks on code availability)

Reviewer #5

(Remarks to the Author)
The author addressed most of my questions, leaving only one remaining: I don’t see a strong reason why this experiment couldn’t be conducted using VR setups.
The author mentions "blinking out of the visual field," which is not a severe issue in most current VR systems, and

"unrealistic spatial trajectories," where minor spatial distortion may occur but generally remains limited. I do not feel that these issues would impact the feasibility of running the experiment in a VR environment. In contrast, for a study focused on foraging behavior, using a screen with mouse and keyboard controls diverges significantly from realistic human behavior. Weighing the pros and cons, conducting this experiment in VR could still offer considerable scientific value, enhancing ecological validity and strengthening the interpretability of the findings. I would encourage the authors to consider revisiting a VR-based setup.

(Remarks on code availability)
The code provides a README file with enough instructions.

Open Access This Peer Review File is licensed under a Creative Commons Attribution 4.0 International License, which permits use, sharing, adaptation, distribution and reproduction in any medium or format, as long as you give appropriate credit to the original author(s) and the source, provide a link to the Creative Commons license, and indicate if changes were made.
In cases where reviewers are anonymous, credit should be given to 'Anonymous Referee' and the source.
The images or other third party material in this Peer Review File are included in the article's Creative Commons license, unless indicated otherwise in a credit line to the material. If material is not included in the article's Creative Commons license and your intended use is not permitted by statutory regulation or exceeds the permitted use, you will need to obtain permission directly from the copyright holder.
To view a copy of this license, visit https://creativecommons.org/licenses/by/4.0/

2 Reviewer 1

2.1 General remarks

R1: Overall, this is very nice work, with an experiment in a virtual foraging environment showing different foraging strategies for smooth (clumpy) and random resource distributions, as well as a difference in asocial and social environments. When foragers were in social environments, they used social cues in the smooth environment when their own performance was poor and other foragers were doing well. The authors use a model competition to identify an area-restricted search strategy that is also conditionally social as the best strategy in smooth and social environments.

I have very few comments on this work as I think it is already in excellent shape.

We thank the reviewer for the thoughtful feedback which has contributed to a much improved manuscript, and for the positive evaluation of our contributions.

2.2 Goldstone and Roberts references

R1: I recall some additional work by Goldstone and Roberts that provides a model of social foraging, EPICURE, and several virtual social foraging experiments. The results provided here are consistent with those, somewhat, but it might be worth discussing them in context to draw out some of the (dis)similarities. Roberts, M. E., & Goldstone, R. L. (2006). EPICURE: Spatial and knowledge limitations in group foraging. Adaptive Behavior, 14(4), 291-313. Goldstone, R. L., Ashpole, B. C., & Roberts, M. E. (2005). Knowledge of resources and competitors in human foraging. Psychonomic Bulletin & Review, 12(1), 81-87.

We thank the reviewer for pointing out these references. We have now cite these and the Goldstone & Ashpole (2004) paper in the introduction and results sections, which we quote below for ease of reference.

Intro
The virtual environment imposes a limited field of view, creating a natural trade-off between allocating visual attention towards individual search or towards peers for social learning (in contrast to Goldstone and Ashpole (2004); Goldstone, Ashpole, and Roberts (2005)).

Results
This emphasizes the need to adapt learning to a dynamically changing information environment, with similar real-world dynamics as developing marketplace innovations or engaging in scientific research (Goldstone et al., 2005; Hills, Todd, Lazer, Redish, & Couzin, 2015; Roberts & Goldstone, 2006).

2.3 Destructive foraging

R1: Social learning can be maladaptive if foraging is destructive as one will simply travel to a location where others have already harvested resources, this is especially true in the random environments. As I understand the results, people were somewhat pulled even in random environments. What might be the explanation here? One explanation is that people did not know that the resources were or were not clustered and this took time to detect? Is there evidence of learning within an environment?

Given the complexity of the study, we wanted to minimize the effect of learning. Hence, we did our best to ensure participants understood the reward structure for both smooth and random environments prior to starting the experiment. This included an interactive tutorial (Supplementary Movie S1: www.youtube.com/watch?v=QksKYOoElxg), where participants were shown a birds-eye illustration of representative environments (similar to the black and white visuals in Fig. 1e and Fig. S14) and were also required to destroy a small group of blocks for each type of environment. In addition, participants completed two practice rounds (asocially) in the different environments to facilitate comprehension of resource distribution. We have emphasized this aspect more clearly now in the main text. Despite our efforts, participants could still have learned about the resource distribution across trials. We tested this using the same hierarchical Bayesian regression as previously reported but adding round as an additional predictor, but found no evidence for any such learning across rounds. This has now been included in the main text.

> **Behavioral results**
> We also did not find increases in foraging success over rounds (random: 0.00 [-0.01, 0.02]; smooth: 0.01 [-0.01, 0.04]), suggesting no substantial meta-learning during the task.

This still leaves the question as to why participants were somewhat pulled to successful others in random environments (Fig. 4d). This indeed is maladaptive in a random environment, but it is possible that such a success-biased copying is hard for people to completely unlearn as it may be deeply ingrained as a strategy as it is beneficial in many environments. Alternatively, people may still have believed that there is some structure present, even in random environments, as people are generally known to see and search for structure, even in random environments. We have added both possible explanations to the discussion.

> **Limitations and future directions**
> Thus, despite resources being distributed randomly, participants were still somewhat drawn towards successful peers. One explanation is that success-biased copying is hard for people to unlearn (e.g., similar to imitation Clay & Tennie, 2018; Lyons, Young, & Keil, 2007), since it is beneficial in many settings. Alternatively, participants may have believed there to be structure in random environments, as is often the case when observing random events (e.g., the gambler's fallacy Hahn & Warren, 2009). Either way, these results suggest potential limitations to the degree of human adaptability and a lingering bias towards social learning.

2.4 Anti-correlated environments

> **R1:** For future work, one could investigate anti-correlated spatial distributions, where targets are not near other targets. Then social learning would be at its worst.

This would indeed be an interesting extension, and we have added this to the discussion on the persistence of success-biased copying in random environments.

> **Limitations and future directions**
> An extreme test could be to use anti-correlated environments to observe if success-biased social learning still persists in the most non-adaptive settings.

2.5 Group problem solving

R1: The authors might want to connect this to the group problem solving literature. Goldstone, R. L., & Gureckis, T. M. (2009). Collective behavior. Topics in cognitive science, 1(3), 412-438. Barkoczi, D., & Galesic, M. (2016). Social learning strategies modify the effect of network structure on group performance. Nature communications, 7(1), 13109

We now connect to these relevant studies in the Discussion.

Discussion
Future work may consider using a non-depleting reward environment, where collective coordination can yield additive benefits to individual search (Barkoczi & Galesic, 2016; Berdahl, Torney, Ioannou, Faria, & Couzin, 2013; Goldstone & Gureckis, 2009; Hawkins et al., 2023)

2.6 Benefits of social foraging

R1: Related to the above, Figure 2a might be interpreted to suggest that social groups are really no better than groups of individuals in this world—harvest rates aren't higher. Perhaps there is a better way to show that, if it's true. That might be related to a notion in Giraldeau, L. A., & Caraco, T. (2000). Social foraging theory (Vol. 73). Princeton University Press, which points out that social groups can often be no better than individuals because as long as they are better, new individuals will join them until the benefits of social foraging are equivalent to solo foraging.

Figure 2a indeed shows that there was no difference in normalized reward rates between individuals and groups. Though we see the connection with the mentioned work on social foraging theory, we decided to not refer to this argument, as we did not allow individuals to join/leave groups. Therefore, although this would be an interesting research question in itself, our study was not designed to test the process of group formation.

However, we have clarified the expectations that the social setting comes with both benefits and costs, in contrast to other commonly used settings without depleting resources.

Results
In contrast to non-competitive contexts (Rendell et al., 2010; Toyokawa, Whalen, & Laland, 2019), where the social setting generally leads to better performance, here peers are both valuable sources of social information (in smooth environments) but also competitors for the same limited resources (Kendal et al., 2018; Laland, 2004) (Fig. S1). This emphasizes the need to adapt learning to a dynamically changing information environment,with similar real-world dynamics as developing marketplace innovations or engaging in scientific research

2.7 Evolutionary generalizability of area-restricted search

R1: The authors might want to point out the evolutionary generalizability of area-restricted search. This isn't entirely relevant on the face of it, but note that area-restricted search can easily be generalized to 'social' resources. People can perseverate on social evidence of resource collection, even when they are not receiving it themselves. This merely requires a method for attaching social evidence of resources to 'reward detection'. I think this complicates and enhances the idea of

area-restricted search and goal-directed cognition I set out here: Hills, T. T. (2006). Animal foraging and the evolution of goal-directed cognition. Cognitive science, 30(1), 3-41. Note in that work that I talk about the evolution of the area-restricted search system to talk about the "expectation of a reward", which can lead to local search around the expectation.

This is an interesting generalization of area-restricted search, which we now touch upon in the discussion. In response to another reviewer comments, we also added one more behavioral results on area-restricted search (Fig. 2b). This figure shows (on a behavioral level) that individuals in smooth (but not random) environments, move further away for the next block after being unsuccessful, while moving shorter distances after finding a reward. We find similar results when looking at turning angles (Fig. S2), while the degree of adaptivity for both foraging distance and turning angles predict individual performance (Figs. S3-S4).

Discussion

A long tradition of research has successfully generalized findings from spatial foraging to more abstract and general settings, such as searching for information on the internet (Pirolli & Card, 1999) or recovering internal memories (Hills, Jones, & Todd, 2012). Since then, recent advances in animal research have incorporated rich behavioral data from visual field analysis (Rahmani, Peruani, & Romanczuk, 2020), spatial trajectories (Sridhar et al., 2021), and network dynamics (Fisher, Ilany, Silk, & Tregenza, 2017) with important new implications, showing how simple (and sometimes seemingly maladaptive) social learning mechanisms can give rise to intelligent behavior in dynamic and ecological environments. Yet, prior to this work, we have lacked behavioral data from humans in an equally immersive setting, which would allow us to account for similar mechanisms. Here, our immersive Minecraft setting has allowed us to address this gap, linking asocial and social mechanisms of adaptive behavior with potential future implications for human social learning beyond a spatial foraging context (Hills, 2006)

3 Reviewers 2 & 3

3.1 General remarks

R2/3: The paper developed a novel Minecraft-inspired collective foraging experiment in an immersive environment to investigate the mechanisms behind adaptive social learning. The task creates a realistic situation in capturing human social foraging behavior and the task design can be an important contribution to the field. The study replicates findings from previous models and theories about foraging behavior and the use of social learning strategies (eg, success biased copy, performance adaptive copying). Although we like the task design, we believe that the motivations behind it and then analyses done leave a few questions unanswered, and some conclusions are not well-supported by the analyses. Mainly, the lack of "adaptive" nature of social learning in this task and comparisons between solo and group foraging, and the general unification of social and asocial learning raise questions.

We thank Reviewers 2 & 3 for the thoughtful and thorough feedback, which has contributed to a substantially improved manuscript. We provide detailed replies below, but as a brief summary, we have:

- Re-written the abstract, introduction, and discussion to redefine goals and contributions
- Performed new analyses on adaptive foraging distance (Fig 2b; reproduced here as Fig. R1) and turning rate (Fig. S2; reproduced here as Fig. R3), which are predictive of individual performance (Figs. S3-S4; reproduced here as Figs. R4-R5)
- Added Fig. 2h (reproduced here as Fig. R1 for ease of reference) to better interpret the methods and results of the temporal analyses
- Added new Bayesian mixed effects regressions, linking model weights to individual performance (Fig. S14; reproduced here as Fig. R2), specifically showing that adaptive social learning weights in group rounds and adaptive asocial weights in solo rounds predict better performance

3.2 General framework and motivations

R2/3: The paper's main motivation is pitched as broad implications for social learning, i.e., what kind of cues underlie adaptive social learning in a foraging context and there is an attempt to distinguish between asocial and social cues, and to unify asocial and social learning in a broad manner. Why is an immersive Minecraft style experiment required to study this? The authors say that the virtual environment poses a limited field of view which creates a natural trade-off between individual search and social learning. Sure, it creates a natural trade-off, but why should we care about visual-spatial dynamics in social learning? For example, in the abstract, the authors start with talking about adaptation to different environments and adaptive behavior and highlight that an integrated framework is missing. They then jump directly to describing the Minecraft environment and the

visual field and spatial data they gain from it. How are visual and spatial data related to adaptive behavior in social and asocial context? Why does visual attention matter?

We are grateful for the reviewers for pointing out this missing link in our argument. A long tradition of research has successfully generalized findings from spatial foraging to more abstract and general settings, such as searching for information on the internet (Pirolli & Card, 1999) or retrieving memories (Hills et al., 2012). It is no accident that these behaviors were first studied in spatial settings and then generalized to other domains. Indeed, in many real-world contexts, space is extremely important and a key mediator of social interactions—from how violent protests erupt in crowds, to how medical teams cooperate, or how human foragers cooperate and compete. In such spatial situations, the environment creates key constraints, such as a trade-off between collecting individual information, or looking towards peers for social information. Yet, these spatial and attentional constraints have yet to be incorporated in research on human social learning.

Meanwhile, animal researchers have recently begun begun to incorporate behavioral data from visual field analysis (Rahmani et al., 2020), spatial trajectories (Sridhar et al., 2021), and network dynamics (Fisher et al., 2017). These results have raised important new implications, showing how simple (and sometimes seemingly maladaptive) social learning mechanisms can give rise to intelligent behavior in dynamic and ecological environments.

Yet, prior to our work, we have lacked behavioral data from humans in an equally immersive setting, which would allow us to study similar mechanisms. And far from being an auxiliary variable, how individuals use their visual attention has immediate repercussions for their own behavior, and the behavior of others, since individuals co-create their own social environment. These fundamental characteristics of many real-world settings are typically stripped away in experiments on social learning, making it unclear how the mechanisms found in such studies translate to more realistic, real-world environments.

Thus, our goal here is use the immersive Minecraft setting to address this gap, with potential future implications for human social learning beyond only spatial environments. To address this popint, we have rewritten the discussion to better clarify this goal (below), while in reply to other related comments (e.g., 3.5) we have also rewritten large parts of the abstract and introduction to clarify our goals and scope. .

Discussion

Our results shed light on the adaptive mechanisms driving collective human behavior, integrating past theories from asocial foraging (Dorfman, Hills, & Scharf, 2022) with context-dependent (Enquist & Ghirlanda, 2007; Morgan, Rendell, Ehn, Hoppitt, & Laland, 2012; Toelch, Bruce, Newson, Richerson, & Reader, 2014) and selective (Garg, Kello, & Smaldino, 2022; Hawkins et al., 2023; Henrich & McElreath, 2003) mechanisms of social learning, which have yet to be combined in a single framework. This integration reveals how these asocial and social mechanisms amplify one another, and are driven by a common currency of individual performance, instead of being governed by separate cues that mutually compensate for one another.

[...]

A long tradition of research has successfully generalized findings from spatial foraging to more abstract and general settings, such as searching for information on the internet (Pirolli & Card, 1999) or recovering internal memories (Hills et al., 2012). Since then, recent advances in animal research have incorporated rich behavioral data from visual field analysis (Rahmani et al., 2020), spatial trajectories (Sridhar et al., 2021), and network dynamics (Fisher et al., 2017) with important new implications, showing how simple (and sometimes seemingly maladaptive) social learning mechanisms can give rise to intelligent behavior in dynamic and ecological environments. Yet, prior to this work, we have lacked behavioral data from humans in an equally immersive setting, which would allow us to account for similar mechanisms. Here, our immersive Minecraft setting has allowed us to address this gap, linking asocial and social mechanisms of adaptive behavior with potential future implications for human social learning (Hills, 2006).

3.3 Adaptive social learning

R2/3: The paper claims that visual spatial information drives adaptive social learning. But at group level, there is no difference in normalized reward rate between solo and group condition. Thus it seems that providing social information does not increase individual foraging performance. Because a person can not individually explore and learn socially at the same time, it is natural for people to switch between social and asocial learning in the group condition compared to solo condition. It remains unclear what is adaptive about social learning. We talk about this point more in the section on Results.

We grateful to the reviewers for raising this important point. We have both clarified the theory in the main text, together with adding new analyses that more clearly establish a link between adaptive social learning and individual performance.

First of all, the equivalent performance between solo and group conditions is not necessarily surprising, because our naturalistic social context provides both *benefits* of social learning via imitation, but also *costs* due to increased competition for shared and depleting resources. This is in contrast to other simpler settings without competition, such as in social multi-armed bandit tasks (Rendell et al., 2010; Toyokawa et al., 2019) where social information is almost always beneficial. However, in order to study adaptive social learning, we specifically required naturalistic dynamics with both benefits and costs for social learning, since a simpler setting where social learning is always beneficial would not impose the same requirements for adaptive learning mechanisms. We have clarified these expectations at the beginning of the results section:

Results
In contrast to non-competitive contexts (Rendell et al., 2010; Toyokawa et al., 2019), where the social setting generally leads to better performance, here peers are both valuable sources of social information (in smooth environments) but also competitors for the same limited resources (Kendal et al., 2018; Laland, 2004) (Fig. S1). This emphasizes the need to adapt learning to a dynamically changing

> information environment, with similar real-world dynamics as developing marketplace innovations or engaging in scientific research (Goldstone et al., 2005; Hills et al., 2015; Roberts & Goldstone, 2006).

Thus, the relevant tests for determining whether there is evidence for adaptive social learning or not does not reside in comparing foraging success of individuals and groups. But rather, in studying how individuals changed their strategies between the four different conditions—particularly in comparing the solo vs. group conditions—and the resulting effects of such strategies. Thus, we have improved our presentation of these results, where our data clearly shows how that adaptive social learning is linked to better individual performance.

Already in our original submission, we reported behavioral signatures of adaptive social learning by showing that visual attention is selectively directed towards successful individuals (Fig. 2k; reproduced here as Fig. R1 for ease of reference) and adapts to individual performance (Fig. 2j), where a lower reward rate is predictive of greater visibility of peers in the future (only in smooth environments). These mechanisms were then implemented in our computational model, where from the estimated weights, we previously reported correlations to a number of behavioral variables, including how greater social adaptivity was correlated with better individual performance.

However, to better emphasize these results, we have now replaced the previous analyses reporting correlations between model weights and individual performance with a Bayesian mixed effects regression approach (Fig. S14; reproduced here as Fig. R2 for ease of reference). Here, the regression simultaneously accounts for all model weights (and for individual and group-level variability as random effects). One key result is that greater adaptivity of success weights (i.e., greater slope of success-biased imitation weight as a function of elapsed time since individual reward) predicts better individual performance (effect in smooth rounds: 2.99 [0.41, 5.58]; Fig. S14). Since other model weights (e.g., asocial mechanisms) are also accounted for in the regression, we can now confidently credit this effect on individual performance to the adaptive mechanisms of social learning.

We are grateful for the reviewers pointing out how this point was not made more clear, and we have now updated the text in several places to i) better communicate the theoretical expectations that a social setting is not expected to only improve performance, and ii) clarify the finding that adpative social learning leads to improvement in individual performance.

> **Behavioral results**:
> Thus, both individuals and groups achieved higher foraging success in smooth environments, whereas the simultaneous benefits and drawbacks of social foraging balanced out at this aggregate level.
>
> **Model weights**:
> Notably, greater social adaptation predicted better performance in group rounds (Adapt. Success: 2.99 [0.41, 5.58]; smooth rounds), while greater asocial adaptation weights predicted better performance in solo rounds (Adapt. Prox: -2.34 [-3.72,-1.03]; smooth rounds).

3.4 Solo vs social

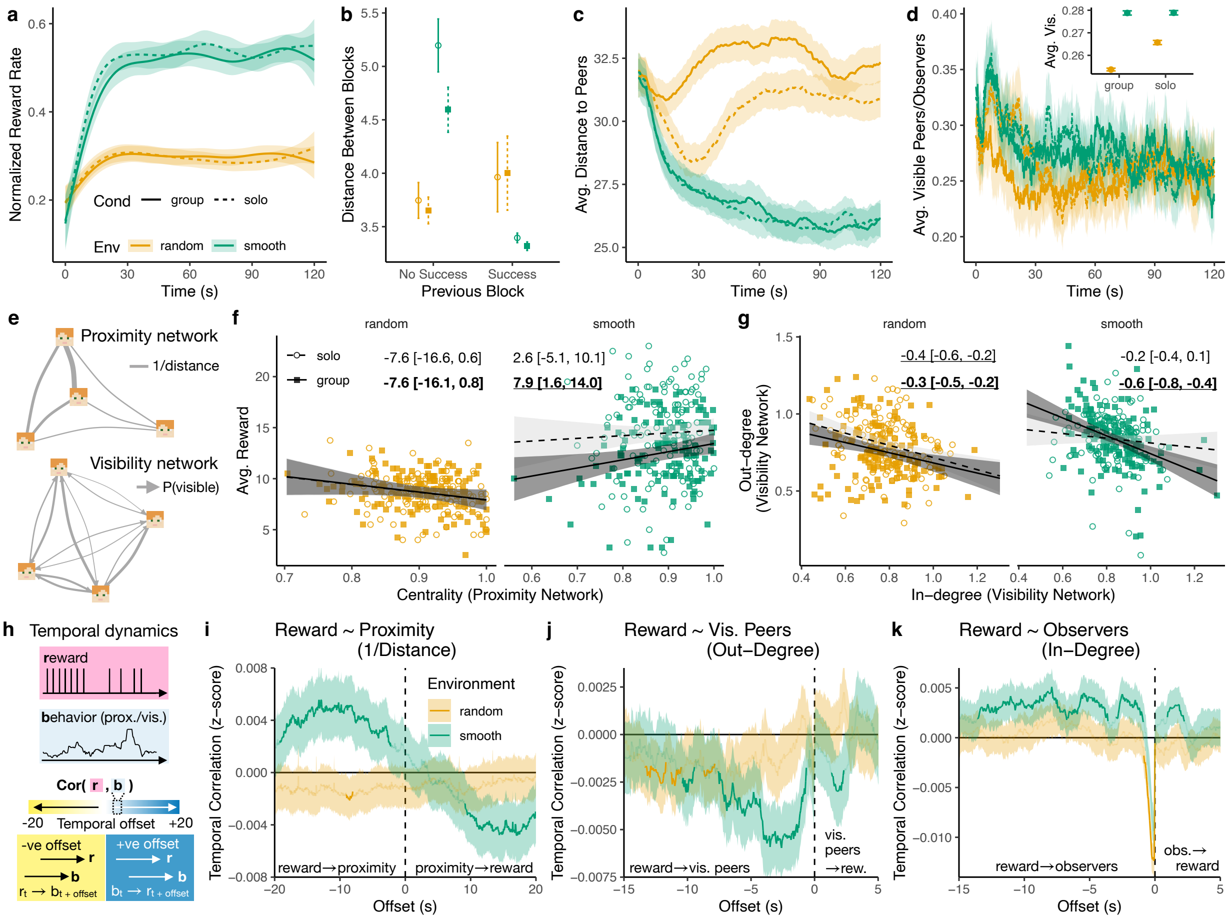

Figure R1: **Behavioral results**. **a**) Normalized reward rate controlling for reward depletion (see Fig. S1). Lines are from a generalized additive model (GAM) for the smoothing of binary data (group means and 95% CI). **b**) Foraging distance as a function of previous success. Dots show the group means, while error bars indicate the 95% CI. **c**) Average distance to peers over time (see Fig. S5a for regression results), with lines showing aggregate means (ribbons are 95% CI). **d**) Average visibility, with the inset showing group means marginalized over time (see Fig. S5b for regression). Note that at the aggregate level, avg. visible peers (outbound) and avg. observers (inbound) are equivalent. (**e-g**) Network analyses. Panel **e** shows examples of proximity and visibility networks. Panel **f** shows average reward as a function of Eigenvector centrality computed on the proximity network. Each dot represents one participant; lines and ribbons show the fixed effect of a hierarchical Bayesian regression (reported above; group rounds in bold). Reliable effects (not overlapping with zero) are underlined. Panel **g** shows the correspondence between in- and out-degree of the visibility network. Each dot represents one participant; the regression line is the fixed effect of a hierarchical Bayesian regression (Fig. S6). (**h-k**) Temporal dynamics of reward rate and proximity/visibility in group rounds (see Fig. S7 for comparison to solo rounds). Panel **h** provides a visual depiction of the analysis (see Methods for details). Results are shown in Panels **i-k**, where the y-axis shows the sign and strength of the correlation (after chance correction). Bold lines indicate significant clusters that survived a permutation analysis (see Methods). Effects at negative offsets indicate that rewards predicts future proximity/visibility, while effects at positive offsets indicate that proximity/visibility predicts future rewards. Positive effects indicate the rewards increased together with behavior, and vice versa for negative effects. Figure 2 in the revised manuscript, reproduced here for ease of access.

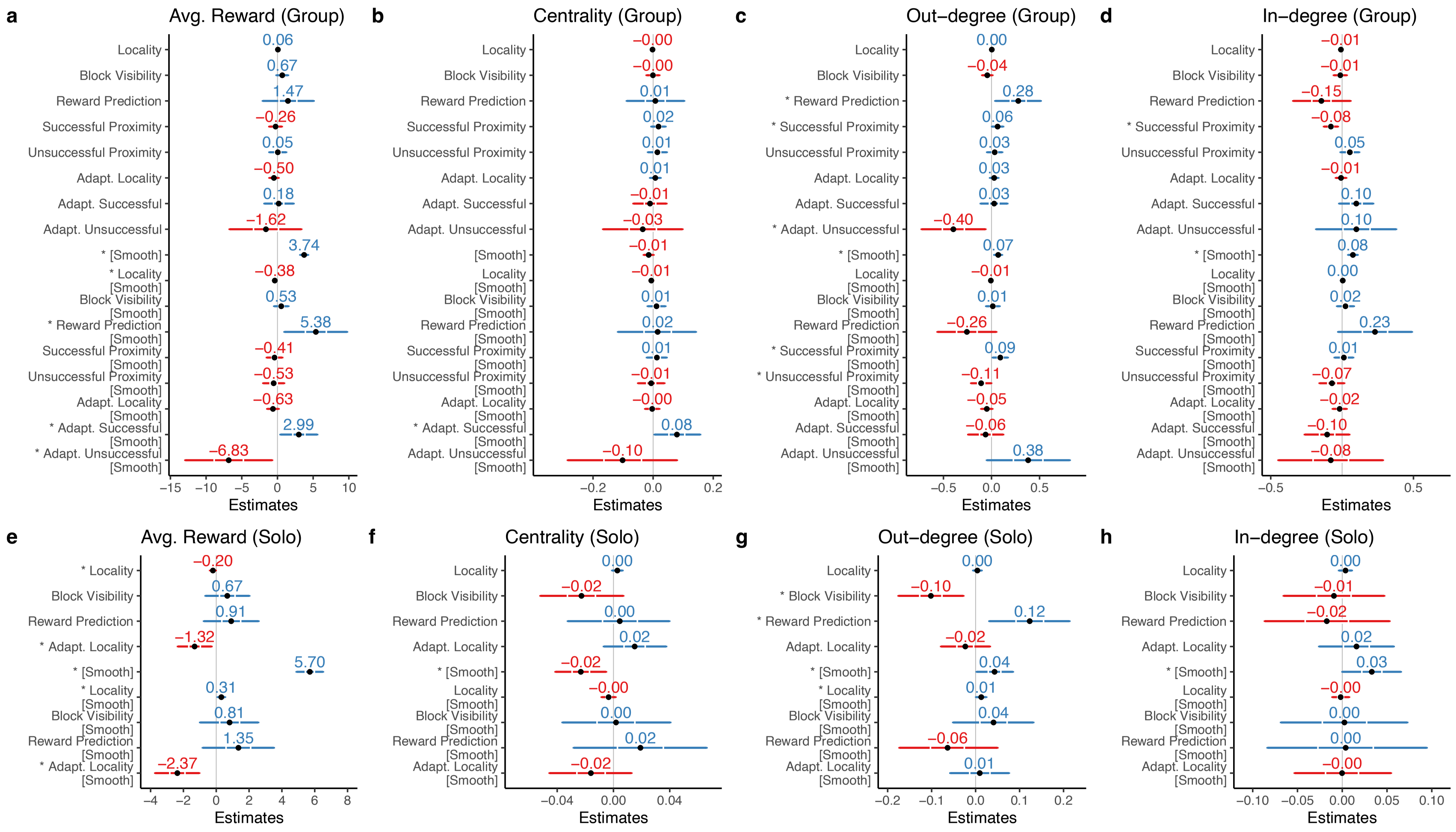

Figure R2: **Regression coefficients of model weights on behavioral variables**. **a-d** are results using the winning ARS+Cond model using data on group rounds, while **e-h** are results for the winning ARS model on solo rounds. All models are hierarchical Bayesian regressions, predicting either average reward (**a**,**e**), centrality (proximity network; **b**,**f**), out-degree (visibility network; **c**,**g**), or in-degree (**d**,**h**), using individual estimates of model weights and their interaction with environment type (random by default, and smooth when indicated). $*$s on the y-axis labels indicate coefficients where the 95% HPD does not overlap with 0. Figure S14 in the revised manuscript, reproduced here for ease of access

> **R2/3:** The authors compared between as-if solo condition and group condition in many (fig a-f) but not all (fig g-i) analyses. We believe some analyses lack controls. In this task, all participants have the same goal and thus in the smooth environment people would gather at the same rewarding location no matter if they were independently exploring or following others. Thus, it is critical to show what effect is driven by social learning and not the environment structure. Please see the later sections for detailed opinions.

We apologize for the confusion on this issue, but the environmental structure was identical across the asocial and social condition, and thus provides exactly the kind of control requested by the reviewers. In the asocial baseline (i.e., solo rounds) each of the four individuals foraged on four exact replications of the same environment (either smooth or random). This allowed us to superimpose the spatial coordinates of each individual, and compute the various measures, such as pairwise distance or visibility as if they were in the same environment. Thus, through comparison to the group rounds, which used the same distribution of smooth or random environments, we could ascribe differences in behavioral measures as being due to social interactions, while behavioral measures that remained the same could be explained away as being due to the structure of the environment. Please note that this asocial baseline was also applied to the temporal analyses, where Figure S4 contrasts the group round effects reported in the main text (Fig. 2i-k) with solo rounds effects. Also, please see our response to comment 3.8a for a related issue regarding the sampling of environments.

We have substantially rewritten our description of the methods and analyses to clarify this key point, and removed the potentially confusing use of "as-if" with scare quotes. We have quoted some selected passages below.

> **Results**:
> In solo rounds, the four participants foraged on separate but identical fields, allowing us to calculate an asocial baseline by superimposing their spatial coordinates as if they were on the same field.
> ...
> Crucially, this pattern is absent in random environments (flat effect) and qualitatively different in the asocial baseline (unimodal positive correlations centered at 0s; see Fig. S4), allowing us to rule out the role of the smooth resource distributions.
> ...
> **Methods: Automated transcription of visual field data**:
> We used a similar procedure for the solo rounds to establish an asocial baseline for our analyses. Whereas all four players searched on different replications of the same field, we simulated the solo as if they were superimposed on the same field.

3.5 Unifying asocial and social

> **R2/3:** a) The paper starts with an ambitious goal of unifying social and asocial theories of learning and decision-making but while the computational model combines social information and personal information, it does not really unify it meaningfully. Many past studies have shown that people balance between social and

asocial information (eg Tump et al 2020, Toyokawa et al 2019) and many models have done so to, so what is this paper's contribution to the "unification"?

b) In addition, the authors refer to ARS for asocial adaptive strategy and critical/conditional social learning as social adaptive strategy and pit them against each other, while they are not really opposing strategies. The basic mechanism of ARS or general optimal foraging theory is related to adapting search state based on the rate of current rewards (or expectation of future rewards). Same goes for critical/conditional social learning, which is based on switching between asocial or social strategy based on reward rate of self (and social partner). They both hinge on the same mechanism, but the only difference lies in where the information about reward is coming from. ARS might as well be called "success-biased" asocial learning because it is biased towards success.

a) We are grateful to the reviewers for pointing out that our key contribution was unclear and that the goal of unification was perhaps too lofty. The reviewers are indeed correct that past research has studied how people integrate and balance social and asocial information, such as in the Tump, Pleskac, and Kurvers (2020) and Toyokawa et al. (2019) papers we cite. However, neither of these are in a foraging context, where peers can be both useful targets for social learning but also competitors for the same limited resources. Thus, mechanisms like ARS that arise from a foraging context are not always appropriate in simple bandit tasks (Toyokawa et al., 2019) or in collective decision-making with individual payoffs (Tump et al., 2020).

Thus, we have removed any reference to unification and refocused clarified our contributions as an integration of social and asocial mechanisms of adaptation that have not previously been brought to bear on the same task.

Abstract

Here, we use a collective foraging task in a virtual Minecraft environment to integrate these two fields, by leveraging automated transcriptions of visual field data combined with high-resolution spatial trajectories[....] These findings not only integrate theories across asocial and social domains, but also provide key insights into the adaptability of human decision-making in complex and dynamic social landscapes.

[...]

Introduction

Adaptive mechanisms have been independently studied in both asocial foraging and social learning, however the two approaches have yet to be integrated in a single framework (Dorfman et al., 2022).

[...]

Goals and scope

Our winning model integrates adaptive mechanisms of asocial and social learning under a single framework, revealing that individual success (rather than social factors) drives changes in asocial foraging patterns and increases both the amount of social learning and selectivity towards successful individuals.

[...]

Discussion

> Our results shed light on the adaptive mechanisms driving collective human behavior, integrating past theories from asocial foraging (Dorfman et al., 2022) with context-dependent (Enquist & Ghirlanda, 2007; Morgan et al., 2012; Toelch et al., 2014) and selective (Garg et al., 2022; Hawkins et al., 2023; Henrich & McElreath, 2003) mechanisms of social learning, which have yet to be combined in a single framework.
>
> [...]
>
> This integration reveals how these asocial and social mechanisms amplify one another, and are driven by a common currency of individual performance, instead of being governed by separate cues that mutually compensate for one another.

b) We agree with the reviewers that ARS and critical/conditional social learning are not opposing strategies, which is why our winning model is indeed a combination of ARS with conditional social learning. To our knowledge (and as reviewed in Dorfman et al., 2022), no other past work has integrated these adaptive asocial and social mechanisms together into a single model. Additionally, there is no clear a priori prediction from the theory that would have suggested that the combination of ARS and conditional social learning would perform best. Indeed, Enquist and Ghirlanda (2007) proposed two forms of adaptive social learning, where the alternative critical social learning (adapting asocial learning depending on social learning success) could have just as easily been the winning model. However, we find that both ARS and conditional social learning together achieve more than the sum of their parts in improving model predictions, thus providing evidence that both asocial and social adaptation are driven by a similar performance-dependent mechanism. We believe this to be a novel and important contribution of our work, which has been further clarified in the discussion

> **Discussion**
>
> Overall, this work integrates previously disparate theories of adaptive mechanisms of asocial and social learning, providing new evidence that they are driven by a common mechanism of individual performance, with their interaction yielding an amplification of adaptivity and selectivity..
>
> [...]
>
> This integration reveals how these asocial and social mechanisms amplify one another, and are driven by a common currency of individual performance, instead of being governed by separate cues that mutually compensate for one another.
>
> [...]
>
> An extreme test could be to use anti-correlated environments to observe if success-biased social learning still persists in the most non-adaptive settings. Indeed, different environments could also yield greater evidence for critical social learning over conditional social learning (Enquist & Ghirlanda, 2007), where costlier asocial learning may prioritize using social cues to adapt learning mechanisms.

We also wanted to comment that one could call ARS "success-biased" asocial learning, although a key difference is that the bias is based on personal performance, whereas the classical use of the term "success-bias" refers to the target of social learning, which is the social peer. Thus, we did not use this term to avoid confusion. However, please also see our reply to comment 2.7, where another reviewer proposed how our work offers a generalization of ARS to a social setting.

3.6 Performance and foraging differences

> **R2/3:** A basic expectation from social learning would have been that players are better at finding rewards with social information. But that is not the case, there is no reliable difference between solo and social condition in the normalized reward rate provided here. What did the overall score look like per participant? Supplementary results say that for smooth environments, participants performed equally well in solo and group condition in terms of normalized reward rate. So how is this adaptive social learning? If there is adaptive social learning indeed, there should be an increase in at least the rate of finding rewards, or in the ratio of reward blocks found / total blocks destroyed. What is the extent of space covered by the participants? If there is no difference in the space covered between social and solo conditions, i.e., they cover the same ground then maybe it can explain why there is no difference in reward rate.

In our reply to comment 3.3, we hope we were able to clarify that the expectation "social is always better" is the case in simple bandit tasks, but not in a foraging/competitive context with depleting rewards. We have clarified this expectation in the introduction, with the changes quoted in response to comment 3.3.

In terms of overall scores per participant and adaptive learning, we have also largely addressed this issue in response to comment 3.3 by clarifying how greater adaptive social learning weights are predictive of better individual performance. However, the questions concerning coverage motivated us to conduct several new analyses that show adaptive changes (i.e., after successful vs. non-successful foraging decisions; Fig. 2b & Fig. S2; the latter is reproduced here as Fig. R3 for ease of reference) in foraging distance between blocks (Fig. S3; reproduced here as Fig. R4) and turning angle (Fig. S4; reproduced here as Fig. R5) predict individual performance in smooth rounds, but not random rounds. These analyses provide preliminary behavioral evidence that helps motivate our inclusion of ARS in our modeling framework, where for our winning model, the adaptivity weights for locality (i.e., ARS mechanism) predicts individual performance in solo rounds (Fig. S14). We include these new results below:

> **Behavioral results**
> Yet, individual performance could be improved through adaptive foraging.To test this hypothesis, we first measured how foraging distance changes depending on whether the previous block yielded a reward (Fig. 2b). In smooth environments, participants foraged more locally after acquiring a reward (-1.6 [-2.0, -1.3]; no effect of group, see Fig. S2a). In contrast, this effect was reversed in random environments, where participants foraged over larger distances after a reward (0.3 [0.1, 0.6]; no effect of group). In turn, greater adaptivity (i.e., difference in foraging distance) predicted better individual performance (Fig. S3) in smooth (solo: $r_\tau = .20$, $p < .001$, $BF = 32$; group: $r_\tau = .18$, $p = .003$, $BF = 9.9$; Kendall's tau) but not random environments (all $p > .05$; $BF < 1$). We obtained equivalent results when measuring the turning angle between blocks (Figs. S2-S4), consistent with the theoretical mechanisms of ARS (Dorfman et al., 2022).
>
> **Model weights**

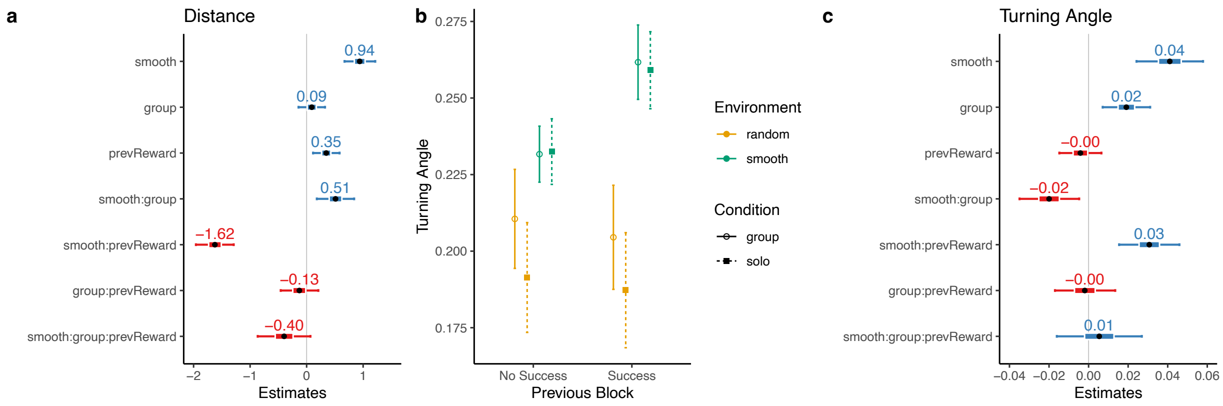

Figure R3: **Adaptive foraging**. **a**) Coefficient plot of a hierarchical Bayesian regression on foraging distance (between blocks). Each dot is the posterior mean and error bars show the 95% CIs. **b**) Turning angle (Eq. **??**) as a function of success. Each dot is a group mean with error bars indicating the SEM. **c**) Coefficient plot of a hierarchical Bayesian regression on turning angle. Figure S2 in the revised manuscript, reproduced here for ease of access.

> [...] greater asocial adaptation weights predicted better performance in solo rounds (Adapt. Prox: -2.34 [-3.72,-1.03]; smooth rounds).

3.7 Visibility differences

> **R2/3:** The authors found that there are no visual differences in the smooth environment between as-if solo and group conditions, but participants reduced social visibility in random environments. This seems to suggest that people use visual spatial information to avoid competition rather than social learning. In addition, the predictor in the regression is undirectional pairwise distance, but later analyses use directional data. So why not separate in-degree and out-degree in this regression analysis?

Figure 2d does indeed shows that there are no differences in the mean number of visible peers in the smooth environment between the solo and group conditions, whereas participants reduced social visibility in random environments (but see the strong solo vs. group differences in visibility in the temporal dynamics analysis; Fig. 2h-k). We also agree that this lends more support for active avoidance of others in the group:random condition, which is also discussed in the social distance results (Fig. 2c). We have now linked these two patterns together more prominently in the results:

> **Behavioral results**
> This analysis revealed greater social distancing in random compared to smooth environments (0.6 [0.8,0.4]; Fig. S5a), which was reliably larger when comparing group rounds to the asocial baseline (0.3 [0.1, 0.5]), suggesting an active increase in avoidance.
> ...
> In contrast, participants reliably reduced social visibility in random environments

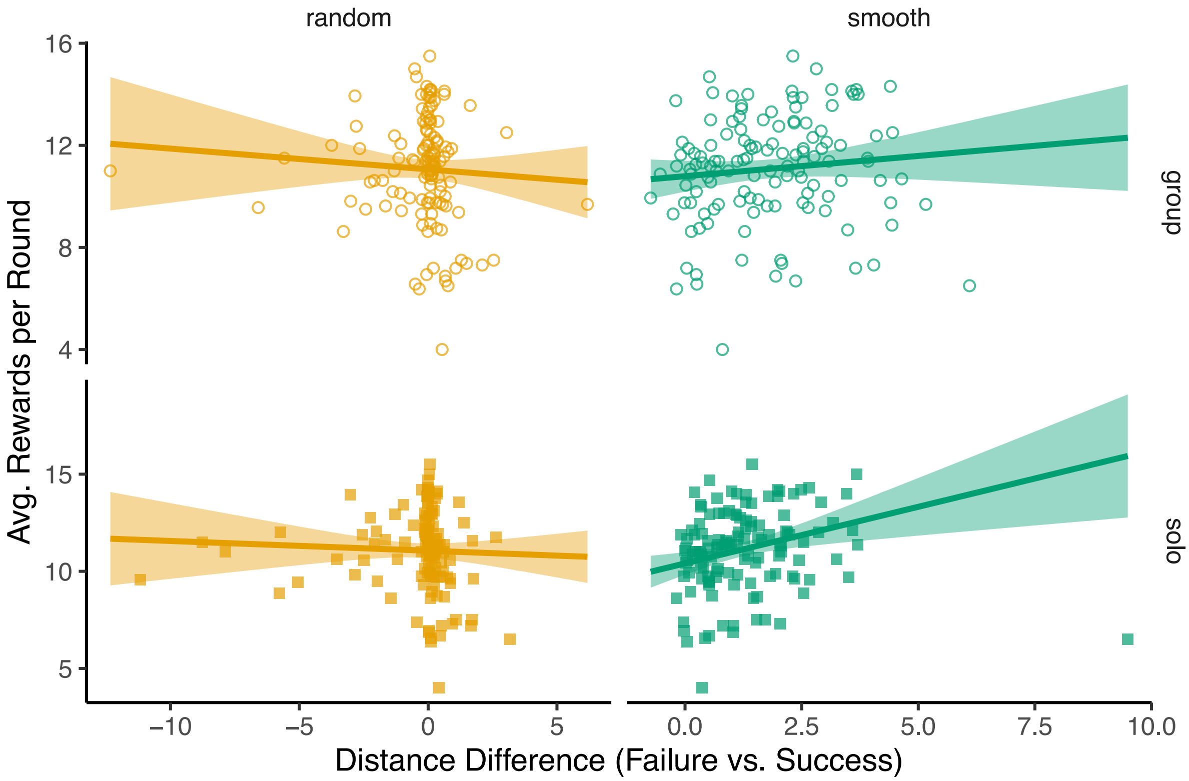

Figure R4: **Adaptive foraging distance and performance**. Adaptive foraging distance is the mean difference of foraging distance (i.e., between consecutive blocks) after a failing to acquire a reward and after successfully acquiring a reward (larger values indicate a decrease of foraging distance after success). Rewards are averaged across the four rounds of each condition. Rewards were correlated to the adaptive distance in smooth (solo: $r_\tau = .20$, $p < .001$, $BF = 32$; group: $r_\tau = .18$, $p = .003$, $BF = 9.9$; Kendall's tau) but not random environments (solo: $r_\tau = -.00$, $p = .993$, $BF = .12$; group: $r_\tau = -.06$, $p = .328$, $BF = .19$). Each dot is a participant, while the lines and ribbons are a linear regression (± 95% CI). A single outlier in smooth:solo was omitted from the linear regression line (around 10 on the x-axis), but not from the rank correlations. Figure S3 in the revised manuscript, reproduced here for ease of access.

compared to the asocial baseline (-0.01 [-0.02, -0.00005]), again showing active avoidance.

Lastly, the question was raised about why some analyses use unidirectional measures, while others use directional measures. The answer is that we started with the most simple approach (often unidirectional), and then moved to more complex analyses when necessary. Indeed, the suggestion of using separate in-degree and out-degree is exactly what the visibility network analysis results in Figure 2g describe (reproduced here as Fig. R1 for ease of reference). Note that this analysis focuses on the relationship between individual in- vs. out- degree, since analysing the overall in- or out-degree (as a function of environment or condition) would simply reduce to the unidirectional regression shown in Figure 2d. We have now changed the text to better communicate this equivalency:

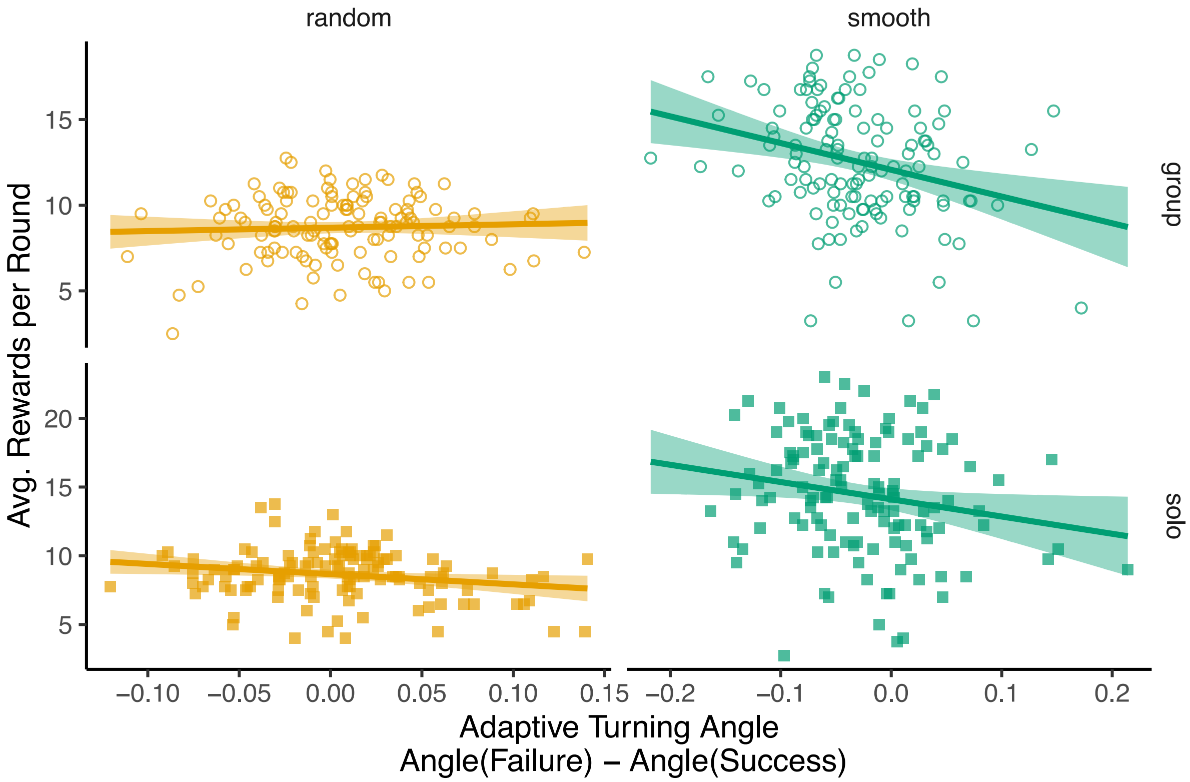

Figure R5: **Adaptive turning angle and performance**. Turning angle (Eq.14) is commonly used to measure foraging trajectories in the context of area restricted search (Dorfman et al., 2022; Pacheco-Cobos et al., 2019), where greater angles correspond to more local search. We measured the mean difference of turning angles after a failing to acquire a reward vs. after successfully acquiring a reward (more negative values indicate a larger increase of turning angle after success, i.e., more local search following success). Rewards are averaged across the four rounds of each condition. Rewards were correlated to the adaptive turning angle in smooth (solo: $r_\tau = -.14$, $p = .020$, $BF = 1.8$; group: $r_\tau = -.18$, $p = .003$, $BF = 10$) but not random environments (solo: $r_\tau = -.08$, $p = .176$, $BF = .30$; group: $r_\tau = .01$, $p = .892$, $BF = .12$). Each dot is a participant, while the lines and ribbons are a linear regression (± 95% CI). Figure S4 in the revised manuscript, reproduced here for ease of access.

Behavioral results
Note that when comparing how visibility changes across conditions at the aggregate level, inbound and outbound social visibility are equivalent, masking individual differences and context-dependent adaptation. We now turn to network analyses to provide a better understanding of the structure and asymmetries of social interactions through visibility

3.8 Asocial baseline

R2/3: a) Can authors clarify how the as-if rounds were constructed? The task structure is a little confusing - there were 16 rounds in the main task, were they

all on different environment structures? Or were there 4 randomly picked environments for each condition (solo vs group, random vs smooth)? Or were there 2 randomly picked environments for random vs smooth, and participants played both solo and group conditions on just these two environments. The "as-if" rounds will only make sense if the environment structure was completely identical between solo and group (for a given resource condition, that is). If that is indeed the case, is there any effect of learning or memory?

b) Plus, why should there be any expectation of meaningful visibility/proximity networks in the as-if models? Better analyses would be to compare how individual foraging behavior changes when in groups – for example through ARS etc. In the smooth environment, there seems to be clear clusters of rewards so do players aggregate on a cluster? One can imagine a cluster of blocks to be a patch and then compare their patch leaving time in solo vs group rounds. In solo foraging, it will be solely based on their "asocial cue" i.e., reward rate, while in group, it will be based on self's reward rate and others' reward rate.

c) It is unclear whether the effects reported here are simply due to the task structure or due to social adaptations. For example, average proximity is higher in smooth rounds than in random rounds. There is a higher likelihood for people to end up on the same cluster of blocks. In addition, due to the solo rounds, they know whether to expect more rewards nearby previous rewards or not based on the color of the blocks. So irrespective of social adaptations or not, they will overlap or not overlap based on the foraging expectations.

a) While a key aspect of this issue was already addressed in response to comment 3.4, here the reviewers raise another key point regarding the sampling of environments. We have now clarified in the methods that participants did not experience any repeat environments in the experiment. Rather, we sampled (without replacement) 8 smooth and 8 random environments (plus 1 additional environment of each type for the practice rounds) from a set of pre-generated environments (Fig S15) containing 20 of each environment type. We have updated the Methods section to make this point more clear.

Participants and design

We generated 20 environments for both smooth and random conditions (Fig. S15), with each session (i.e., group) sampling without replacement 1 (practice) + 8 (main task) = 9 environments of each class with pseudorandom assignments that were pregenerated prior to the experiment.

Thus, while solo and group rounds were not performed on the exact same environment, they were sampled from the same stationary distributions for each environment generating process. Although the reviewers suggest that a comparison between solo and group rounds requires the environment to be exactly identical, we believe that this would be less reliable and introduce a critical confound. If participants foraged over the same environments in both solo and group rounds, there could be a learning effect, since the second time in the same environment would confer advantages. And since round order could only be counterbalanced across groups of participants, this would make it impossible to compare solo vs. group rounds at the individual level. However, we do not currently observe any learning over rounds (without repeating environments), which is an important sanity check to ensure our analyses across environments and between conditions is valid. We have now added this null result in the text:

Behavioral results
We also did not find increases in foraging success over rounds (random: 0.00 [-0.01, 0.02]; smooth: 0.01 [-0.01, 0.04]), suggesting no substantial meta-learning during the task.

b) We hope that our clarifications about how we computed the asocial baselines ("as-if" rounds) in reply 3.4 should have already addressed this issue, since we are able to uniquely identify which changes in behavior are due to the environment (comparing rough vs. smooth) and which changes are due to the social structure (solo vs. group). Additionally, our new analyses on adaptive foraging distance and turning angle (Figs. 2b, S2-S5) are quite similar to the suggested new analyses, thus providing early behavioral evidence for ARS and converging towards the same interpretation as the rest of our analyses.

c) The result that the reviewers point out about how people are closer together in smooth rounds is a good example of how we can use our 2x2 design to distinguish environmental vs. social effect. We do not see any difference in social proximity between solo vs. group rounds for smooth environments, where participants in solo rounds are foraging individually on replicated versions of the same environment. Thus, we can conclude that the social condition did not lead to any aggregate changes in social proximity. However, as later analyses looking at social structure and temporal dynamics reveal, there are unique social effects (not found in solo rounds) such as greater centrality predicting better individual rewards (Fig. 2f) and success-dependent spatial cycling (Fig. 2i).

3.9 Proximity and visibility networks

R2/3: a) Are the network weights static and averaged over the whole experiment or are they dynamic?

b) Visibility networks: The authors found an asymmetry between the in- and out-degree are more in the group+smooth sections. How does an inverse relationship suggest "specialization of social learning strategies"? What is the strategy that is being discussed and what does it mean for it to be specialized? This effect can also be driven by the spread of players, if there are some players who are more ahead of others or moving in a different direction then others can see them but they can't see others much. Alternatively, this could also be driven by the fact that in general some participants looked more in the group section and thus other participants got more social attention. This would explain the effect of out- and in-degree in random environments and not just in smooth. In smooth, this would lead to a stronger effect because most people are localized near each other and the few people who are away from the rest of the group will have a high in-degree. In random, because people are dispersed and not synchronized the effect is weaker.

a) Sorry for not clarifying this point. Network weights are static and and computed over rounds, which are then averaged over participants for our statistical analyses. We have clarified this in the results section

Proximity and visibility networks
Network weights are computed per individual per round, and then averaged per

individual (within each condition), with the same analyses also applied to solo rounds to provide an asocial baseline.

b) Thank you for raising some interesting hypotheses regarding these results. Here, specialization refers to the fact that rather than clustering together in the center of Fig. 2g with similar in- and out- degrees, they become spread to the extremes as either strongly individual learners with high in-degree but low out-degree (i.e., “producers”), or as strongly social learners with low in-degree and high out-degree (i.e., “scroungers”). Thus, your first hypothesis about how some players who move ahead ahead of the pack and are thus not observed, would be consistent with this story. We have now updated the text to clarify this interpretation:

> **Proximity and visibility networks**
> This suggests an increased specialization of social learning strategies (i.e., “producers” with low out-degree and high in-degree or “scroungers” with high out-degree and low in-degree) and asymmetry of social attention in settings where social information was useful.

However, your second hypothesis is inconsistent with our data, since the group setting did not increase social visibility overall (Fig. 2d). Rather, the visibility networks suggest only an increase in specialization, where some individuals became more strongly social and some became more strongly individual, while at the aggregate level, the amount of social visibility remained the same. The hypothesis of there still being an effect in the random environment is intriguing, but runs contrary to the statistics, since it is a very robust null result, and is further corroborated by consistent social vs. asocial differences in the temporal dynamics (Fig. 2h-k), pull analyses (Fig. 3) and computational modeling results (Fig. 4). Nevertheless, we have de-emphasized the importance of this specialization result, since it is not such a central part of our findings.

3.10 Temporal analyses

> **R2/3:** a) The authors need to better explain the clusters and correlations and offsets, it is hard to follow. For example, what are “chance corrections”? What are predictive clusters and how were they chosen?
> b) Again, why will the as-if condition show any effect of proximity and reward temporality? A better comparison would be to see what individual players do when they have low reward rate and high reward rate in solo vs group. If the behavior (movement, head turns or something) is different between solo and group, only then there would be clear proof that players socially adapted the strategy. The authors mention ARS but then it is only discussed in the computational results. The “performance-adaptive-social-cycling” is attributed to social adaptation: poor rewards lead to increased future proximity and high rewards lead to decreased future proximity. While we agree that people follow a player who has found something, the “social-cycling” need not be purposeful – the destructive blocks will inadvertently lead to that effect because after exhausting the rewards in an area (high rewards), the strategy can be to move away from that area (like in ARS) and proximity reduction will be a side-effect. Then when they see someone else find a reward, they converge to a local area. Is there a social and asocial comparison to get at the behavior?

c) Similarly for adaptive social attention, low rewards predicted seeing more peers in the immediate future and vice-versa, this can be very well driven by the destructive structure and the expectation of reward structure which will lead them to move away or look away after destroying enough rewards in the local area. How do head turns or rotations in social rounds compare with solo because that is the meaningful comparison.

a) We are grateful for the reviewers for pointing this out, and have now added Figure 2h as a visual description about how the analyses are performed and how to interpret the results. We have also further clarified the meaning of chance corrections and predictive clusters in the main text and methods sections.

Temporal dynamics
More precisely, we computed correlations between time-series at different temporal offsets (Fig. 2h), and searched for temporally continuous clusters of significance (indicated by bold lines in Fig. 2i-k) that survived multiple forms of chance correction (see Methods). At negative offsets, these clusters indicate that rewards predicts predicts future proximity/visibility (either positive or negative correlations depending on the sign), while positive offsets indicate that proximity/visibility predicts future reward.

Methods: Temporal dynamics
Each correlation was then z-transformed and corrected for chance using a permutation baseline (i.e., permutation chance correction). This chance correction is based on iteratively permuting the order of `V2` and computing the correlation `cor(V1,V2_permuted)` over 100 different permutations (for each correlation). We then subtracted the z-transformed mean of the permutation correlations from the target correlation. These permutation corrected correlations are reported as a population-level mean ($\pm$95% CI) in Figure 2i-k and Figure S7. [...]

Lastly, to provide better interpretability of these results, we used a maximum cluster mass statistic (Nichols & Holmes, 2002) to discover temporally continuous clusters of significance at the population level. For each pair of variables $[V1,V2]$ and within each combination of condition (solo vs. group) and environment (random vs. smooth), we used a cluster permutation test to find a threshold for random clusters. This analysis used 10,000 permutations, where for each, we iterated over each individual time series of z-transformed (and chance-corrected) correlations, randomly flipping the sign at each time point. We then used a single-sample t-test with $\alpha = .05$ to compute which time points (at the population level) were significantly different from 0. This provided a distribution of the duration of temporally continuous clusters of significance in the randomly permuted data. We then used the upper 95% CI of this distribution as a minimum threshold for the actual data, where we applied the same significance testing procedure, but discarded all clusters shorter in duration than the permutation threshold. The surviving clusters are illustrated with bold lines in Figure 2i-k and Figure S7.

Please also note that we identified and fixed a bug with the previously reported analyses. They have been corrected in Figure 2i-k and we have rewritten the corresponding interpretation of the results, which we quote. However, the main story of the manuscript remains unchanged.

Temporal dynamics

First, the dynamics of individual reward (i.e., foraging success) and spatial proximity to other players revealed a pattern of *success-dependent spatial cycling* in smooth (but not random) environments (Fig. 2i). The positive correlation at offset -19s to -2s (bold line) indicates that greater reward predicted greater proximity at long timescales into the future. Since previous analyses showed that success corresponded to reduced foraging distances (Fig. 2b) and increased turning angles (Fig. 2b), the greater proximity predicted by reward is more likely due to attracting peers when successful. Subsequently, this creates a cyclical pattern, where the negative correlation at offset 9s to 20s indicates that greater proximity predicts lower future rewards, likely due to depletion. Lower rewards then drives reduced proximity (i.e., reward→proximity effect at negative offsets), with reduced proximity driving increased rewards, thus continuing the cycle. Crucially, this pattern is absent in random environments (flat effect) and qualitatively different in the asocial baseline (unimodal positive correlations centered at 0s; see Fig. S7), allowing us to rule out the role of the smooth resource distributions. These dynamics reveal the process behind why greater centrality was correlated with performance (Fig. 2f), showing that success temporarily attracts imitators, who then dissipate when the resources are depleted.

Next, we looked at the dynamics of reward and visual field data, where we analyzed the number of visible peers (outbound social attention towards others; Fig. 2j) and the number of observers (inbound social attention; Fig. 2k) at every timepoint. Starting with outbound visibility of peers (Fig. 2j), we found evidence for *adaptive social attention*. First, we found several negative correlation clusters at negative offsets for both environments, indicating that low rewards predicted greater visual attention towards others (and vice versa). However, only the cluster in smooth environments survives (from -2.85s to -0.45s) when computing the contrast between group and solo rounds (Fig. S7). Thus, our comparison to the asocial baseline allows us to identify that social attention adapts to individual performance, above and beyond the generic effects of the task structure (i.e., reduced visibility when destroying a block). Additionally, we also observe opportunity costs of social attention in smooth rounds (1.3s to 2.3s), where the negative correlation indicates that more visible peers predicted lower rewards. This effect is absent in random environments and reversed in the asocial baseline (Fig. S7), which accounts for the shared reward structure, indicating it is a distinctly social phenomenon.

Lastly, the dynamics of reward and inbound visibility (number of observers) indicate *success-biased selectivity*, where participants who acquired higher rewards were the target of social attention (Fig. 2i). In smooth environments, we observed two clusters at negative offsets (-13.6s to -3.7s and -3.0s to -1.0s) with positive correlations, indicating that greater rewards predicted more observers in the future. This success-biased selectivity was absent in random environments and inverted in the asocial baseline (Fig. S7). The strong dip approaching offset = 0s is due to the visual dynamics of the task, since the splash animation temporarily obscured the avatar when acquiring a reward. Additionally, we observe another positive correlation cluster at offsets 1.0s to 2.2s in smooth environments, indicating having

more observers predicted greater future rewards. This effect was again absent in random environments and inverted in the asocial baseline (Fig. S7).

In sum, these temporal dynamics reveal how individual performance is the key driver of adaptive mechanisms, driving changes in social proximity (Fig. 2i) and social attention—both when (Fig. 2j) and towards whom (Fig. 2k) it is directed.

b) We hope we have clarified the asocial baseline comparisons in response to previous comments. The new Figure 2b analyses we conducted in response to comment 3.6 now provides exactly the analysis requested, about how foraging distance and turning rate change as a function of reward rate, across both environments and for solo vs. group.

Here, the reviewers raise an interesting hypothesis regarding our adaptive cycling result in Figure 2i, suggesting that it might be solely due to block destruction that drives the effect rather than purposeful social imitation. It is indeed true that in the temporal dynamics analysis of reward∼proximity, we are not able to pinpoint the specific mechanisms that drive this affect. However, subsequent analyses show that participants display purposeful increases in social visibility as a function of lower reward (Fig. 2j), are more likely to be "pulled" in leader-follower dynamics (Fig. 3), and are selectively reducing their distance to successful individuals in our trial-by-trial modeling of foraging decisions (Fig. 4)—with each analysis surviving comparison to an asocial baseline. Thus, the overwhelming evidence suggests that this is purposeful adaptation to the social setting.

c) Here, these concerns can be addressed by controlling for the asocial baseline, where despite having the exact same reward structure and task dynamics (i.e., destroying blocks impacting visibility), we still see a reliable effect. We quote the updated text below for clarification:

> **Temporal dynamics**
> First, we found several negative correlation clusters at negative offsets for both environments, indicating that low rewards predicted greater visual attention towards others (and vice versa). However, only the cluster in smooth environments survives (from -2.85s to -0.45s) when computing the contrast between group and solo rounds (Fig. S7). Thus, our comparison to the asocial baseline allows us to identify that social attention adapts to individual performance, above and beyond the generic effects of the task structure (i.e., reduced visibility when destroying a block).

Additionally, the reviewers asked about head turns or rotations. While we have added an analysis of turning rate (Fig. S2 and S4), these are necessarily computed across multiple block destruction events for stability (following Pacheco-Cobos et al., 2019) and to avoid sensitivity to overly noisy measurements. Thus, we believe that our current analyses of peer visibility provide the most effective and consequential way to address these questions, and which have already been utilized across all levels of analysis in the manuscript: total visibility (Fig. 2d), bidirectional visibility network analysis (Fig. 2g), temporal dynamics (Fig. 2j-k), and computational modeling of trial-by-trial choices (Fig. 4).

3.11 Computational modeling

R2/3: a) The comparisons between behavioral results and the model seem disjointed. We'd like to see more connections of the model to the behavioral results

(eg, what distinctive behavioral patterns do different models predict?) and other visibility analyses (eg, does the model predict more pull behavior / more in degree / ect?). In addition, ARS was used in the model but there is no discussion in the behavioral results.

b) The winning model combines ARS and conditional social learning, which hints at the precedence of individual success rate in deciding the next step. However, this may not be as general an effect as the paper makes it out to be. In environments where individual search is costlier or when there are no prior expectations from the environment, critical social learning would be more adaptive. The effect of task design in the "general" features of adaptive behavior need to be specified.

a) We are grateful to the reviewers for their suggestions which have allowed us to make more concrete connections between our behavioral results and our models. First, we have added new adaptive foraging distance (Fig. 2b) and turning rate analyses (Fig. S2) that both predict individual performance (Figs. S3-S4) and provide initial evidence for ARS-like mechanisms. Additionally, the spatial cycling pattern (Fig. 2h) motivates our decision to implement social mechanisms based on proximity-based imitation. And lastly, the success-driven selectivity of social attention from the visibility network (Fig. 2g), temporal dynamics (Fig. 2k), and pull analyses (Fig. 3d) all help motivate us distinguishing between successful vs. unsuccessful social proximity in the models. We have now modified the text in numerous locations to better clarify these connections.

Behavioral results
Yet, individual performance could be improved through adaptive foraging. To test this hypothesis, we first measured how foraging distance changes depending on whether the previous block yielded a reward (Fig. 2b). In smooth environments, participants foraged more locally after acquiring a reward (-1.6 [-2.0, -1.3]; no effect of group, see Fig. S2a). In contrast, this effect was reversed in random environments, where participants foraged over larger distances after a reward (0.3 [0.1, 0.6]; no effect of group). In turn, greater adaptivity (i.e., difference in foraging distance) predicted better individual performance (Fig. S3) in smooth (solo: $r_\tau = .20$, $p < .001$, $BF = 32$; group: $r_\tau = .18$, $p = .003$, $BF = 9.9$; Kendall's tau) but not random environments (all $p > .05$; $BF < 1$). We obtained equivalent results when measuring the turning angle between blocks (Figs. S2-S4, consistent with the theoretical mechanisms of ARS (Dorfman et al., 2022).

Behavioral summary
Overall, participants *adaptively* deployed both asocial and social learning mechanisms according to the environment and depending on individual performance, and *selectively* directed social attention towards successful individuals. These behavioral results provide a lens into the dynamics of how asocial and social learning interact and feedback onto each other. However, they only indirectly speak to the individual-level cognitive processes that drive decision making in our experiment. Therefore, we next turn to computational models integrating different combinations of asocial and social learning mechanisms to predict individual foraging decisions.

Social features
We then incorporate social features based on proximity to different subsets of play-

ers, motivated by our results showing selective visual attention (Fig. 2j) and a susceptibility to being "pulled" (Fig. 3d) towards successful individuals.

Model comparison

ARS is based on past work using area-restricted search (Dorfman et al., 2022; Tinbergen, Impekoven, & Franck, 1967), and uses only asocial features, but with the locality weight changing as a function of time since the last individual reward. This corresponds to the common finding (Hills et al., 2015) that search distance adapts to foraging success (Fig. 2b), and was the best model in solo rounds and the second best in group rounds (Fig. 4b-c)

b) We agree with the reviewers about potential limits to this interpretation, where different environments or different tasks may support a critical social learner over a conditional social learner. The hypothesis that the relative costs of individual vs. social learning may be the driving factor behind which form of learning is considered first sounds very appealing, and we have incorporated this suggestion into the general discussion:

Limitations and future directions

An extreme test could be to use anti-correlated environments to observe if success-biased social learning still persists in the most non-adaptive settings. Indeed, different environments could also yield greater evidence for critical social learning over conditional social learning (Enquist & Ghirlanda, 2007), where costlier asocial learning may prioritize using social cues to adapt learning mechanisms.

3.12 Minor comments

R2/3: a) The authors wrote "Whereas high centrality tended to correspond to low rewards in random environments (group: -7.6 [-16.6,0.6]; solo: -7.6 [-16.1,0.8]; all slopes overlap with 0)". Although the authors used the word "tended", I don't think they can draw any conclusion from a confidence interval overlapping with 0.
b)The introduction can be better set up. Many terms are dropped in without context or explanation of what they mean. For example, what social or asocial cues or factors should we expect in the following sections? What does "influence and selectivity" of social learning mean?
c)The papers cited in the first line of Discussion do not really refer to collective foraging as a metaphor for social learning to my knowledge.

a) Thank you for pointing this out. The intention was to indicate this as a null effect, but also to highlight that the overall direction was reversed compared to our main effect (which was reliable). We have now rewritten it to be more clear:

Whereas centrality did not predict rewards in random environments (group: -7.6 [-16.6,0.6]; solo: -7.6 [-16.1,0.8]; all slopes overlap with 0), we found a robust inversion in smooth environments, where higher centrality predicted higher rewards in group rounds (7.9 [1.6,14.0]; Fig. 2f)

b) Together with the more general comments and feedback from other reviewers, the introduction has been substantially rewritten. Specifically we have rewritten the section using "influence and selectivity" of social learning to be more clear:

> **Goals and scope**
> Our winning model integrates adaptive mechanisms of asocial and social learning under a single framework, revealing that individual success (rather than social factors) drives changes in asocial foraging patterns and increases both the amount of social learning and selectivity towards successful individuals. Furthermore, it is the degree of adaptivity—of both asocial and social learning—that best predicts individual performance.

However, we believe it would be misleading to introduce the different asocial and social "cues" in the introduction, since the different aspects of the model correspond to "mechanisms". Using the concept of cues, may mislead the reader to expect them to come from different modalities or variables, whereas these are often overlapping. For instance, both visibility (asocial) and successful vs. unsuccessful proximity (social) are defined based on the visual field transcription, whereas the latter social mechanisms also integrate spatial location as well.

Thus, we believe that introducing ARS and conditional/critical social learning mechanisms in the introduction provides the correct theoretical framing. Whereas other mechanisms, such as selectivity and locality are motivated through the early behavioral analyses.

c) We have modified the citations in the discussion to more directly, together with references suggested by the other reviewers. We have kept however, the Hills et al., (2015) paper due to the numerous references to social and spatial foraging.

> Collective foraging is a common metaphor for human social learning (Garg et al., 2022; Hills et al., 2015; Roberts & Goldstone, 2006).

4 Reviewer 4

4.1 General remarks

> **R4:** Upfront, I'm a statistician with no expertise in the content area of the paper. My area of expertise is hierarchical modeling and I was asked to review those aspects of the paper. To be frank, although I am passively familiar with agent-based modeling and network modeling, I don't have much expertise in this area and am not familiar with nuances and best practices, so I am not sure how relevant or informative my review will be.
>
> That said, I thought the detail on the hierarchical models looked pretty good. I thought the number of MCMC iterations (4,000) looked a little low for this kind of model, but the authors did a convincing job reporting convergence/diagnostic information on p. 13 to demonstrate that the results are reasonably trustworthy. The models were also described completely and it was generally clear to me how things were set up.

We thank Reviewer 4 for their thoughtful comments on our statistical analyses. The number of MCMC iterations was chosen to ensure sufficient coverage of the posterior distribution while minimizing computational costs. Note that, even with massive parallel evaluation of the likelihood on a computing cluster, each of those models took several days to finish. However, as noted by the reviewer, all convergence diagnostics confirm that the number of iterations was sufficient for reliable inference.

4.2 Random effects

> **R4:** As a moderately important comment, the description on p. 11 says the random effects in the model control for participant characteristics. This is a common misconception, but it is not actually true. The random effects serve to partition the unexplained variance into different sources (e.g., within-unit vs between-unit), but the variance is still unexplained and participant characteristics are not conditioned out. If you truly wanted to control for participant characteristics, you'd have to use a fixed effect approach (e.g., include dummy variables for each unit) to absorb the participant sources of variance or you'd have to center the predictors in a way that make the within-unit and between-unit submodels orthogonal such that participant characteristics could not affect within-unit associations (e.g., a within-between specification where within-unit predictors are unit-mean centered and the unit-mean is included as a between-unit predictor; e.g., Bell & Jones, 2015, Political Science Research & Methods; McNeish & Kelly, 2019, Psychological Methods). To be clear, I'm not saying that the model is wrong or inappropriate, just that I don't believe the description matches what the model is doing.

Thank you for this comment. We agree that the previous language implied a strict sense of causal "control" for unobserved participant- and group-level characteristics, which our models cannot necessarily achieve. Therefore, throughout the text (see below), we now only speak of multilevel models "accounting for" participant-/group-level variability, which does not imply that we strictly *explain* such variability, only that it is partitioned out in the model.

Results: Computational modeling of choices
Block features **f** capture hypotheses about individual and social learning mechanisms (see below), while weights **w** are estimated using hierarchical Bayesian methods, accounting for individual and group variability as random effects (see Methods).

Methods: Hierarchical Bayesian regressions
Statistical analyses were conducted using hierarchical Bayesian regressions to simultaneously model the effects of the experimental manipulations (smooth vs. random and solo vs. group), while accounting for participant- and group-level variability.

4.3 LKJ prior

R4: As some minor comments, it'd be helpful to report the argument for the LKJ prior (typically referred to as eta). I'm guessing it was 1 since that is commonly used so that the prior is a random correlation matrix of a particular dimension, but different arguments for LKJ correspond to correlation matrices with different properties that may not necessarily be diffuse/non-informative.

We apologize for omitting this important information. We used weakly informative LKJ priors with shape parameter $\eta = 4$ for all random-effect correlation matrices, which put less weight on extreme correlations of 1 or -1 (see Fig. R6 for reference). We have now also clarified that we used standard normal priors for feature weight parameters and exponential priors with $\lambda = 1$ for the random-effect standard deviations:

Methods: Computational modeling
To minimize the risk of overfitting the data, we used weakly informative priors for all parameters. We used weakly informative standard normal priors centered on 0 for all weight parameters, exponential priors for scale parameters (with rate parameter $\lambda = 1$) and LKJ priors (with shape parameter $\eta = 4$) for correlations matrices (Lewandowski, Kurowicka, & Joe, 2009).

4.4 CIs

R4: Additionally, CIs are mentioned in a few places but it is not defined (confidence intervals or credible interval? CI can be used for both). If credible interval, it would be useful to mention what kind of interval (e.g., symmetric, highest posterior density). P.2 mentions highest density intervals, but it wasn't clear if this referred to just those values or to any CI throughout the paper and in the figures.

Results in the main text are reported as posterior means and 95% Highest Posterior Density Intervals. We have now also added this information in the Methods section.

Methods: Hierarchical Bayesian regressions
Effects are reported as the posterior mean and 95% Highest Posterior Density Interval.

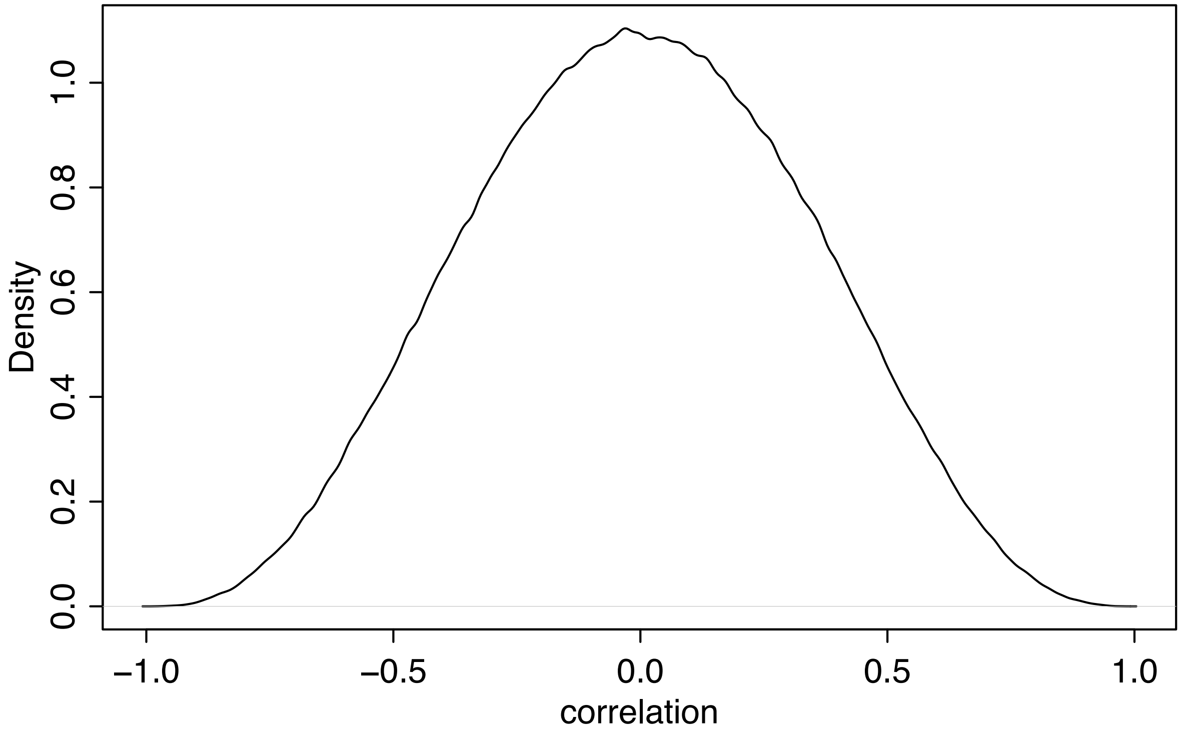

Figure R6: **LKJ prior for correlation matrices.**

5 Reviewer 5

5.1 General remarks

R5: The paper aims to elucidate the mechanisms of social and asocial learning within a unified framework. The study integrates both non-social and social learning strategies within a virtual Minecraft environment, where participants engage in collective foraging tasks. Automated transcription of visual field data and high-resolution spatial trajectories are used to analyze social interaction dynamics. The paper demonstrates that people dynamically adapt their non-social and social learning mechanisms based on environmental conditions and personal performance, specializing their social attention patterns and selectively directing social learning toward successful individuals. Additionally, the study introduces a computational modelling framework that integrates various combinations of non-social and social learning mechanisms to predict individual foraging decisions sequentially.

The primary strength of this paper lies in its practical experimental framework, which allows for a comparative analysis of social and asocial learning. The use of a Minecraft-based foraging game is particularly clever, as it enables participants to engage in both learning strategies and provides a rich experimental setup that mirrors real-world social interactions. This framework facilitates dynamic anal-

> ysis of decision-making processes at each time point. By quantifying abstract human learning strategies through experimental results, the study establishes a solid foundation for future experimental modelling. Incorporating computational models adds depth to the behavioural analysis, offering a new understanding of cognitive processes. This methodological approach provides significant insights and inspiration for future research, demonstrating a multidimensional strategy that could have a profound impact on social science studies.

We thank the Reviewer for their insightful comments which have improved the manuscript, and their positive evaluation of our work.

5.2 Theoretical Contributions

> **R5:** The paper's theoretical contributions are not clearly articulated. The experimental results are somewhat broad and scattered, making it unclear what key scientific question the authors are addressing. Are they attempting to show that individuals can balance social and asocial learning based on their goal of maximizing personal performance? This may not be a novel finding, as from an evolutionary perspective, striving for optimal performance is a natural expectation for enhancing foraging efficiency. It gives the impression that, despite the authors employing new designs and conducting extensive data analyses and modeling, these efforts have not yielded new scientific insights for the field. In other words, the authors should clarify how their approach addresses gaps left by existing theoretical and experimental frameworks and what significant theoretical conflicts have been resolved through their methodology.

We are grateful for the reviewers comments, which have been immensely helpful in improving how we communicate our theoretical contributions. The reviewer is correct in identifying our contribution as understanding how individuals balance social and asocial learning with the goal of maximizing personal performance. However, our results reveal novel insights about the dynamics of adaptive learning and the specific mechanisms driving this balance, which cannot simply be derived from existing evolutionary approaches.

Specifically, our analyses provide a novel understanding of i) how the dynamic adaptation of social and asocial mechanisms stem from a shared criterion based on personal performance, and ii) the degree of this dynamic adaptation is what predicts individual performance, rather than other predictors of behavior that omit these dynamics.

To better clarify our contributions, we have rewritten the introduction and discussion to better reflect these arguments:

> **Goals and scope**
> By combining visual field analysis with high-resolution spatial trajectories, we provide new perspectives about how asocial and social learning mechanisms complement one another in a dynamic and integrative fashion. Our results show that people dynamically *adapt* asocial and social learning mechanisms to both the environment and individual performance and *selectively* direct social learning towards successful individuals (Fig. 2). Our behavioral analyses capture both the structure and temporal dynamics of social interactions (Fig. 2-3), which are then directly

tested using computational models sequentially predicting each foraging decision (Fig. 4). Our winning model integrates adaptive mechanisms of asocial and social learning under a single framework, revealing that individual success (rather than social factors) drives changes in asocial foraging patterns and increases both the amount of social learning and selectivity towards successful individuals. Furthermore, it is the degree of adaptivity—of both asocial and social learning—that best predicts individual performance.

Discussion

Our results shed light on the adaptive mechanisms driving collective human behavior, integrating past theories from asocial foraging (Dorfman et al., 2022) with context-dependent (Enquist & Ghirlanda, 2007; Morgan et al., 2012; Toelch et al., 2014) and selective (Garg et al., 2022; Hawkins et al., 2023; Henrich & McElreath, 2003) mechanisms of social learning, which have yet to be combined in a single framework. This integration reveals how these asocial and social mechanisms amplify one another, and are driven by a common currency of individual performance, instead of being governed by separate cues that mutually compensate for one another.

[...]

Notably, individual estimates of adaptivity of both asocial (i.e., ARS) and social learning mechanisms (i.e., success-biased imitation) predicted better individual performance (Figs. S3,S4, & S14). Overall, this work integrates previously disparate theories of adaptive mechanisms of asocial and social learning, providing new evidence that they are driven by a common mechanism of individual performance, with their interaction yielding an amplification of adaptivity and selectivity.

5.3 Leadership

R5: While the analysis of leadership effects on social learning is interesting, its relevance to the paper's main argument—that individual performance drives the adaptivity of both local foraging and selective social learning—is unclear. The implications of leadership positions on social attention and behaviour are not well-integrated into the core thesis. This aspect might be more appropriately explored as a separate study to better understand its significance and impact.

We believe the leadership aspect is important because it identifies concrete events where a person was drawn to another individual. In the other behavioral results (i.e., Fig. 2), we present proxies/correlates of possible social information use (e.g., visual attention or proximity to others), but the leadership analysis identifies concrete incidences where an individual's foraging trajectory was influenced by another. Hence, we believe it plays an important role in our argument on social learning, where we can relate leadership scores to these other behavioral measures (Fig. S9). We have now clarified prior to the leadership section what role this analysis plays in the paper. Moreover, we now draw the link between pulls in the leadership events (Fig. 3), and weighting of successful others in the computational modeling (Fig. 4d), which approximate the same phenomenon (i.e., approaching successful others), to clarify the links between the behavioral results and the computational modeling.

Temporal dynamics
We next relate these social attention processes to social interactions characterized by concrete leader-follower dynamics.

Computational modeling of choices: Social features
We then incorporate social features based on proximity to different subsets of players, motivated by our results showing selective visual attention (Fig. 2j) and a susceptibility to being "pulled" (Fig.3d) towards successful individuals.

5.4 Gaze vs. Heading

R5: The paper should distinguish between "gaze" and "heading." In real-world scenarios, gaze and heading are not always aligned due to differences in eye orientation, head direction, and body orientation. Since participants in the study used a screen and mouse and keyboard controls, their gaze and heading are likely to be consistent, which does not fully reflect real-world conditions. Although the authors chose this approach to avoid VR motion sickness, it may lead to discrepancies between participants' actual walking trajectories and the avatars' movements. Given that data collection likely began in 2019 (as indicated by the ethics approval number) and considering the advancements in VR technology over the past five years, including improvements that mitigate motion sickness, modern all-in-one VR headsets now allow users to walk freely in an unlimited virtual space. Incorporating this technology could significantly enhance the naturalness of the experiment by enabling participants to walk within the virtual environment while interacting with the game. I recommend that the authors utilize VR headsets that support free movement in virtual spaces to improve the experiment's realism and potentially achieve more accurate results.

We are grateful for these comments. First of all, we have replaced all mention of gaze with heading to be more accurate.

Secondly, as mentioned in the Methods section, we originally designed the experiment for use with VR headsets. Indeed, we even tested the experiment using multiple forms of VR locomotion technology, including with harnesses and a slippery walking surface (and special shoes or shoe covers) to allow for unrestricted locomotion. However, each of these methods had significant drawbacks, as is evident by their lack of adoption in a commercial setting. The VR headsets we used were HTC Vive headsets, which are still relatively state-of-the-art, and the most common form of unlimited locomotion that avoids VR motion sickness is to use teleportation over short distances. However, by design, this results in a blinking out of the visual field (to avoid the feeling of acceleration) and also produces unrealistic spatial trajectories. Thus, we really exhausted all available VR options before deciding that our research questions would be best served using desktop computers to provide the most realistic medium to capture visual and spatial data in an immersive foraging setting.

Although we hope new advances in technology will allow us to address related research questions in a truly VR setup, we nevertheless believe that our experiments is able to provide important scientific contributions while also raising the bar in terms of realism and immersion.

5.5 Concept of Social Interaction

> **R5:** The paper should clarify what is meant by "social interaction," as it is a broad concept. It appears to use an abstract notion rather than specifying whether it refers to direct face-to-face interaction or verbal communication. The authors should clearly define what "social interaction" specifically represents in their study.

We are grateful for this suggestion. We define "social interactions" via the various methods we can measure them, based on spatial distance, visibility, and detecting leader-follower dynamics in our "pull" analyses. To clarify this multi-faceted approach, we have modified the text to indicate that each of these analyses are designed to analyze social interactions, but that none of them is wholly sufficient:

> **Behavioral results**
> Next, as an initial analysis of social interactions, we computed the average pairwise distance between participants across conditions (Fig 2c).
>
> [...]
>
> Note that when comparing how visibility changes across conditions at the aggregate level, inbound and outbound social visibility are equivalent, masking individual differences and context-dependent adaptation. We now turn to network analyses to provide a better understanding of the structure and asymmetries of social interactions through visibility
>
> **Temporal dynamics**
> We next relate these social attention processes to social interactions characterized by concrete leader-follower dynamics.

5.6 Data Analysis Justification

> **R5:** Given the complexity of the data analyses, the authors should provide more details at the beginning of each results section about the necessity of each analysis. This includes explaining the rationale behind each analysis, the hypotheses being tested, and the insights that the results reveal.

We are grateful for this suggestion and have now changed the text throughout the results section to clarify the main aim of different analyses, along with a new Figure 2h panel that visually explains how we conducted the temporal dynamics analysis. We quote some of these changes below:

> **Behavioral results**
> To understand how the environment and social interactions impacted performance, we examine the normalized reward rate (Fig. 2a), which controls for faster reward depletion in group rounds with more individuals searching for the same number of finite rewards (see Fig. S1).
>
> [...]

Yet, individual performance could be improved through adaptive foraging. To test this hypothesis, we first measured how foraging distance changes depending on whether the previous block yielded a reward (Fig. 2b).

[...]

Next, as an initial analysis of social interactions, we computed the average pairwise distance between participants across conditions (Fig. 2c).

[...]

We first used the proximity network to compute the Eigenvector centrality for each participant, providing a holistic measure of the influence each node exerts on the network.

Temporal dynamics

To analyze the dynamics of social interactions, we searched for temporally predictive clusters relating individual reward to our high-resolution spatial-visual data (Fig. 2h-k; see Methods). More precisely, we computed correlations between time-series at different temporal offsets (Fig. 2h), and searched for temporally continuous clusters of significance (indicated by bold lines in Fig. 2i-k) that survived multiple forms of chance correction (see Methods). At negative offsets, these clusters indicate that reward predicts future proximity/visibility (either positive or negative correlations depending on the sign), while positive offsets indicate that proximity/visibility predicts future reward.

5.7 Closing remarks:

R5: In sum, while the paper presents a sophisticated experimental design and innovative methodology, it needs to clarify its theoretical contributions and ensure that all aspects of the research, including leadership analysis and technology choices, are well-justified and contribute to the main thesis. Addressing these concerns will strengthen the paper and its impact on the field of social and asocial learning research.

We hope that our replies above and the associated changes in the text have clarified these important issues, and agree that addressing these has helped improve the manuscript.

References

Barkoczi, D., & Galesic, M. (2016). Social learning strategies modify the effect of network structure on group performance. *Nature communications*, *7*(1), 13109.

Berdahl, A., Torney, C. J., Ioannou, C. C., Faria, J. J., & Couzin, I. D. (2013, Jan). Emergent sensing of complex environments by mobile animal groups. *Science*, *339*(6119), 574–576. Retrieved from `http://dx.doi.org/10.1126/science.1225883` doi: 10.1126/science.1225883

Clay, Z., & Tennie, C. (2018). Is overimitation a uniquely human phenomenon? insights from human children as compared to bonobos. *Child development*, *89*(5), 1535–1544.

Dorfman, A., Hills, T. T., & Scharf, I. (2022). A guide to area-restricted search: a foundational foraging behaviour. *Biological Reviews*, *97*(6), 2076–2089.

Enquist, M., & Ghirlanda, S. (2007). Evolution of social learning does not explain the origin of human cumulative culture. *Journal of Theoretical Biology*, *246*(1), 129–135.

Fisher, D. N., Ilany, A., Silk, M. J., & Tregenza, T. (2017). Analysing animal social network dynamics: the potential of stochastic actor-oriented models. *Journal of Animal Ecology*, *86*(2), 202–212.

Garg, K., Kello, C. T., & Smaldino, P. E. (2022). Individual exploration and selective social learning: balancing exploration–exploitation trade-offs in collective foraging. *Journal of the Royal Society Interface*, *19*(189), 20210915.

Goldstone, R. L., & Ashpole, B. C. (2004). Human foraging behavior in a virtual environment. *Psychonomic bulletin & review*, *11*(3), 508–514.

Goldstone, R. L., Ashpole, B. C., & Roberts, M. E. (2005). Knowledge of resources and competitors in human foraging. *Psychonomic Bulletin & Review*, *12*(1), 81–87.

Goldstone, R. L., & Gureckis, T. M. (2009). Collective behavior. *Topics in cognitive science*, *1*(3), 412–438.

Hahn, U., & Warren, P. A. (2009). Perceptions of randomness: why three heads are better than four. *Psychological review*, *116*(2), 454.

Hawkins, R. D., Berdahl, A. M., Pentland, A. Tenenbaum, J. B., Goodman, N. D., & Krafft, P. (2023). Flexible social inference facilitates targeted social learning when rewards are not observable. *Nature Human Behaviour*, *7*(10), 1767–1776.

Henrich, J., & McElreath, R. (2003). The evolution of cultural evolution. *Evolutionary Anthropology: Issues, News, and Reviews: Issues, News, and Reviews*, *12*(3), 123–135.

Hills, T. T. (2006). Animal foraging and the evolution of goal-directed cognition. *Cognitive science*, *30*(1), 3–41.

Hills, T. T., Jones, M. N., & Todd, P. M. (2012). Optimal foraging in semantic memory. *Psychological review*, *119*(2), 431.

Hills, T. T., Todd, P. M., Lazer, D., Redish, A. D., & Couzin, I. D. (2015). Exploration versus exploitation in space, mind, and society. *Trends in cognitive sciences*, *19*(1), 46–54.

Kendal, R. L., Boogert, N. J., Rendell, L., Laland, K. N., Webster, M., & Jones, P. L. (2018, July). Social learning strategies: Bridge-Building between fields. *Trends in Cognitive Sciences*, *22*(7), 651–665. doi: 10.1016/j.tics.2018.04.003

Laland, K. N. (2004, February). Social learning strategies. *Learning & behavior*, *32*(1), 4–14. doi: 10.3758/bf03196002

Lewandowski, D., Kurowicka, D., & Joe, H. (2009). Generating random correlation matrices based on vines and extended onion method. *Journal of Multivariate Analysis*, *100*(9),

1989–2001.
Lyons, D. E., Young, A. G., & Keil, F. C. (2007). The hidden structure of overimitation. *Proceedings of the National Academy of Sciences*, *104*(50), 19751–19756.
Morgan, T. J. H., Rendell, L. E., Ehn, M., Hoppitt, W., & Laland, K. N. (2012, February). The evolutionary basis of human social learning. *Proceedings of the Royal Society B: Biological Sciences*, *279*(1729), 653–662. doi: 10.1098/rspb.2011.1172
Nichols, T. E., & Holmes, A. P. (2002). Nonparametric permutation tests for functional neuroimaging: a primer with examples. *Human Brain Mapping*, *15*(1), 1–25.
Pacheco-Cobos, L., Winterhalder, B., Cuatianquiz-Lima, C., Rosetti, M. F., Hudson, R., & Ross, C. T. (2019). Nahua mushroom gatherers use area-restricted search strategies that conform to marginal value theorem predictions. *Proceedings of the National Academy of Sciences*, *116*(21), 10339–10347.
Pirolli, P., & Card, S. (1999). Information foraging. *Psychological review*, *106*(4), 643.
Rahmani, P., Peruani, F., & Romanczuk, P. (2020). Flocking in complex environments—attention trade-offs in collective information processing. *PLoS computational biology*, *16*(4), e1007697.
Rendell, L., Boyd, R., Cownden, D., Enquist, M., Eriksson, K., Feldman, M. W., … Laland, K. N. (2010). Why copy others? insights from the social learning strategies tournament. *Science*, *328*(5975), 208–213.
Roberts, M. E., & Goldstone, R. L. (2006). Epicure: Spatial and knowledge limitations in group foraging. *Adaptive Behavior*, *14*(4), 291–313.
Sridhar, V. H., Li, L., Gorbonos, D., Nagy, M., Schell, B. R., Sorochkin, T., … Couzin, I. D. (2021). The geometry of decision-making in individuals and collectives. *Proceedings of the National Academy of Sciences*, *118*(50), e2102157118.
Tinbergen, N., Impekoven, M., & Franck, D. (1967). An experiment on spacing-out as a defence against predation. *Behaviour*, *28*(3-4), 307–320.
Toelch, U., Bruce, M. J., Newson, L., Richerson, P. J., & Reader, S. M. (2014, February). Individual consistency and flexibility in human social information use. *Proceedings of the Royal Society B: Biological Sciences*, *281*(1776), 20132864. doi: 10.1098/rspb.2013.2864
Toyokawa, W., Whalen, A., & Laland, K. N. (2019). Social learning strategies regulate the wisdom and madness of interactive crowds. *Nature Human Behaviour*, *3*(2), 183–193.
Tump, A. N., Pleskac, T. J., & Kurvers, R. H. (2020). Wise or mad crowds? The cognitive mechanisms underlying information cascades. *Science Advances*, *6*(29), eabb0266.

1 Reviewer 1

1.1 General remarks

> **R1:** I'm very satisifed with the revision. It's sophisticated and enlightening and demonstrates a nice potential interaction between social and asocial area-restricted search. The next step will be to see if they share underlying mechanisms. How nice it would be to show that the evolution of spatial search is somehow related to social learning and mental search more generally, and perhaps shares fundamental dimensions.

We are very grateful to the reviewer for the valuable feedback which has greatly improved our manuscript. We also share the same enthusiasm for these future questions about the evolution of search strategies that we hope our contributions may help unlock.

2 Reviewers 2 & 3

2.1 General remarks

> **R2/3:** We thank the authors for addressing our comments, especially by tying their motivations closer to the experiment and results. We agree that the integration of asocial foraging and social information is novel, and this new virtual foraging paradigm is a good way to study this question. The comparison with as-if solo rounds is also explained more clearly in this revision.

We thank Reviewers 2 & 3 for their detailed feedback, which has helped us greatly improve the motivations and clarity of the presentation of our results.

2.2 Greater emphasis on contributions

> **R2/3:** The authors started from the assumption that these are adaptive and showed that people employed these computations in social foraging, and employed them differently in different environments. And the computational models provide a proof for this assumption. The authors also showed that greater adaptive weights predict better performance in social foraging. We think this is a major finding supporting the main motivation and title, and could be emphasized more.

We are very glad the reviewers found these to be major findings, and we have now tweaked the manuscript to improve our emphasis on these factors.

> **Results: Computational modeling of choices**
> Thus adaptivity of both social and asocial learning mechanisms is what predicts individual performance.
>
> **Discussion**
> Furthermore, it is the degree of adaptivity—for both social and asocial learning mechanisms—that best predicts individual performance.

2.3 Mechanisms and effects

R2/3: The authors also talked about how people with greater centrality performed better, and leaders achieved higher instantaneous reward. But these are effects instead of mechanisms. We hope these could be tied more to the main motivation, or addressed in the Discussion.

We thank the reviewers for raising this issue, which we have now clarified in the manuscript.

Behavioral results: Temporal dynamics
These dynamics reveal the mechanisms behind why greater centrality was correlated with performance (Fig.2f), showing that success temporarily attracts imitators, who then dissipate when resources are depleted.

3 Reviewer 4

3.1 General remarks

R4: I was Reviewer 4 on the original submission. All of my comments related to the statistical analyses were satisfactorily addressed and I have no further comments on this version of the paper.

We thank Reviewer 4 for their helpful comments in clarifying our statistical analyses.

4 Reviewer 5

4.1 Remaining question about VR

R5: The author addressed most of my questions, leaving only one remaining: I don't see a strong reason why this experiment couldn't be conducted using VR setups. The author mentions "blinking out of the visual field," which is not a severe issue in most current VR systems, and "unrealistic spatial trajectories," where minor spatial distortion may occur but generally remains limited. I do not feel that these issues would impact the feasibility of running the experiment in a VR environment. In contrast, for a study focused on foraging behavior, using a screen with mouse and keyboard controls diverges significantly from realistic human behavior. Weighing the pros and cons, conducting this experiment in VR could still offer considerable scientific value, enhancing ecological validity and strengthening the interpretability of the findings. I would encourage the authors to consider revisiting a VR-based setup.

We thank the Reviewer for their valuable comments which have greatly improved the manuscript. Regarding this remaining question, we added more clarification about this point in the discussion (moving some text from the methods).

Discussion: Limitations and future directions.

Our task was originally designed for data collection using a virtual reality (VR) headset instead of a mouse and keyboard. However, preliminary testing revealed that locomotion via teleportation (the preferred method to avoid VR motion sickness) resulted in less naturalistic

spatial trajectories and interfered with visual field analyses due to the field of view temporarily fading to black during movement. Several VR treadmill products were also tested as an alternative form of locomotion, but required substantial training and resulted in large individual differences in navigation ability. In contrast, the current computer-based modality captured naturalistic trajectories in both space and heading direction. However, future studies should continue to strive for greater ecological validity using new advances in VR.
